# Grain-Filling Rate Improves Physical Grain Quality in Barley Under Heat Stress Conditions During the Grain-Filling Period

**DOI:** 10.3389/fpls.2022.858652

**Published:** 2022-05-13

**Authors:** Hamid Shirdelmoghanloo, Kefei Chen, Blakely H. Paynter, Tefera Tolera Angessa, Sharon Westcott, Hammad Aziz Khan, Camilla Beate Hill, Chengdao Li

**Affiliations:** ^1^Department of Primary Industries and Regional Development, Northam, WA, Australia; ^2^School of Molecular and Life Sciences, Curtin University, Perth, WA, Australia; ^3^Department of Primary Industries and Regional Development, Perth, WA, Australia; ^4^Western Crop Genetics Alliance, College of Science, Health, Engineering and Education, Murdoch University, Perth, WA, Australia

**Keywords:** grain weight, plumpness, heat stress, grain-filling, stay-green, water-soluble carbohydrates, barley

## Abstract

Heat stress is a primary constraint to Australia's barley production. In addition to impacting grain yield, it adversely affects physical grain quality (weight and plumpness) and market value. The incidence of heat stress during grain filling is rising with global warming. However, breeding for new superior heat-tolerant genotypes has been challenging due to the narrow window of sensitivity, the unpredictable nature of heat stress, and its frequent co-occurrence with drought stress. Greater scientific knowledge regarding traits and mechanisms associated with heat tolerance would help develop more efficient selection methods. Our objective was to assess 157 barley varieties of contrasting genetic backgrounds for various developmental, agro-morphological, and physiological traits to examine the effects of heat stress on physical grain quality. Delayed sowing (i.e., July and August) increased the likelihood of daytime temperatures above 30°C during grain-filling. Supplementary irrigation of field trials ensured a reduced impact of drought stress. Heat tolerance appeared to be the primary factor determining grain plumpness. A wide variation was observed for heat tolerance, particularly among the Australian varieties. Genotypic variation was also observed for grain weight, plumpness, grain growth components, stay-green and stem water-soluble carbohydrates (WSC) content, and mobilisation under normal and delayed sown conditions. Compared to normal sowing, delayed sowing reduced duration of developmental phases, plant height, leaf size, head length, head weight, grain number, plumpness, grain width and thickness, stem WSC content, green leaf area retention, and harvest index (HI), and increased screenings, grain length, grain-filling rate (GFR), WSC mobilisation efficiency (WSCME), and grain protein content. Overall, genotypes with heavier and plumper grains under high temperatures had higher GFR, longer grain-filling duration, longer green leaf area retention, higher WSCME, taller stature, smaller leaf size, greater HI, higher grain weight/plumpness potentials, and earlier flowering. GFR played a significant role in determining barley grain weight and plumpness under heat-stress conditions. Enhancing GFR may provide a new avenue for improving heat tolerance in barley.

## Introduction

Barley (*Hordeum vulgare* L.) is an important temperate cereal crop best adapted to environments with an optimum temperature of 15°C during grain-filling to achieve maximum grain mass (Chowdhury and Wardlaw, 1978). However, in many growing regions, including the Australian grain belt, the barley crop is frequently exposed to high-temperature damage (days above 30°C) during flowering and grain development (Wardlaw and Wrigley, 1994; Asseng et al., 2011). During flowering and grain development, high-temperature damage can be classified as acute or chronic. Acute damage results from short exposure to high temperatures (heat shock and typically temperatures above 35°C), while chronic damage results from exposure to elevated temperature for a long duration during flowering and grain development (heat stress and typically temperatures above 30°C). The increasing frequency and severity of heat extremes predicted due to climate change are likely to challenge global food security in the future (Asseng et al., 2011; Xie et al., 2018).

Heat stress causes an average of 15% yield loss p.a. in temperate cereals in Australia (Wardlaw and Wrigley, 1994; Telfer et al., 2013). Using a panel of 138 barley genotypes, a study in Denmark reported a 56% grain yield reduction caused by heat stress (Ingvordsen et al., 2015). While most studies report reductions in grain yield, heat stress dramatically reduces barley grain weight and plumpness and increase grain size inconsistency (Macnicol et al., 1993; Savin and Nicolas, 1996). The resultant downgrading of malt barley to feed can cause a significant loss of market value for barley growers. Heat stress, therefore, has severe implications for the future of the Australian grain crop industry and highlights an urgent need to develop heat-tolerant barley varieties.

Heat stress during meiosis leads to floret sterility and, consequently, failed seed set (Saini and Aspinall, 1982; Sakata et al., 2000). At the early grain-filling stage, heat reduces final grain weight and size (Macnicol et al., 1993; Savin and Nicolas, 1996). Direct selection for heat tolerance is difficult due to the apparent narrow window of sensitivity, the unpredictable nature of heat stress (i.e., timing, magnitude, and duration), and its frequent co-occurrence with drought stress. Therefore, a greater scientific knowledge regarding traits and mechanisms associated with heat tolerance (and its genetic variability) would help develop more efficient selection methods.

A range of physiological and biochemical processes are adversely affected by high temperatures, resulting in reduced grain yields and quality (Cossani and Reynolds, 2012). These processes, exclusively and in combination, could potentially represent the basis for genotypic variation in heat tolerance. Approximately 70% of the barley grain mass consists of starch, which is the grain component most diminished under high-temperature conditions, and its synthesis depends on both the supply of assimilates to the developing grain and their conversion into starch within the grain (Wallwork et al., 1998; Shirdelmoghanloo et al., 2016b). Heat stress accelerates the rate of senescence and leaf chlorophyll loss, leading to reduced photosynthetic capacity and assimilate supply to the developing grains. The ability to retain green leaf area during grain-filling (stay-green) under stress conditions would help assimilate supply and lead to a higher rate and a longer duration of grain-filling under such conditions. Maintenance of grain weight under field and controlled conditions for heat and drought are often associated with the stay-green trait in wheat (Kumari et al., 2007; Lopes and Reynolds, 2012; Shirdelmoghanloo et al., 2016b,c) and sorghum (Borrell et al., 2014a,b). Water-soluble carbohydrate (WSC) reserves in the stems and leaf sheaths serve as an alternative source of assimilates, significantly buffering against the loss of green area and photosynthetic capacity during reproductive stages under heat and drought stress (Blum, 1998). Stem WSC content is dynamic and is the net outcome of deposition, remobilisation, and losses caused by other processes (e.g., respiration). Talukder et al. (2013) reported a positive association between stem WSC mobilisation and the heat stability of grain-filling in wheat. Evidence on the contribution of WSCs to variability in the ability of barley genotypes to maintain grain weight and size under heat stress is scarce.

Nevertheless, mobilised WSCs can be estimated from the difference between peak and minimum WSCs content during grain-filling. Sensitivity of processes within or close to the developing grain may also be crucial factors. These processes include heat sensitivity and recovery of lost activity following heat relief of several enzymes in the starch biosynthesis pathway of the developing grain (Wallwork et al., 1998) and accelerated maturation of the grain by heat stress, provoked by stress signals such as ethylene (Hays et al., 2007). Studying these traits in a diverse range of barley genotypes exposed to natural heat stress could explain the underlying tolerance mechanisms and drivers of better physical grain quality under heat stress and genetic variability. Shirdelmoghanloo et al. (2016a) suggested that this would allow complementary selection criteria when breeding for heat tolerance.

For experimental purposes, heat stress can be mimicked in the field by late sowing, covering plots with tunnels, or using in-field heat chambers. Under more controlled conditions, temperature-controlled growth rooms or glasshouse compartments can be used to grow plants and transfer them at specific developmental stages to defined heat stress treatments (Borghi et al., 1995; Savin and Nicolas, 1996; Passarella et al., 2008; Talukder et al., 2013; Thistlethwaite et al., 2020). However, a greater emphasis should be placed on field responses when breeding for heat tolerance to relate the relevance of the results to the barley industry (Passioura, 2006).

The present study measured physiological processes, phenology, and plant architecture in a diverse set of 157 barley genotypes. The aim was to gain insights into their relative contributions to physical grain quality stability, genetic variability, and their interactions under heat stress conditions during the grain-filling period. Barley plants were sown later than standard farming practise to ensure heat stress coincided with grain-filling. Such an approach allows the evaluation of large numbers of lines for heat tolerance under field conditions (Abou-Elwafa and Amein, 2016; Sissons et al., 2018; Thistlethwaite et al., 2020).

## Materials and Methods

### Germplasm

In 2016 and 2017, a collection of flowering date and grain plumpness characteristics of 300 barley (*Hordeum vulgare* L.) genotypes occurred across various environments (unpublished data). A subset of 157 two-row barley genotypes was selected for this study's detailed phenotyping (Supplementary Table S1). The genotypes selected comprised released varieties, advanced breeding lines, and landraces, with 106 originating from Australia, 18 from North America, 11 from Europe, nine from South Africa, eight from South America, three from the International Center for Agricultural Research in the Dry Areas (ICARDA), and two from Asia. Seven lines were only screened in one location (Supplementary Table S1).

### Field Experiments

Wongan Hills (WH) and Muresk (MK) in Western Australia were the locations of the field trials in 2018 and 2019 (Table 1). At WH, three planting dates represented normal (NS), late (LS), and very late sowing (VLS) conditions, while there was only LS at MK. The LS and VLS increased the probability of heat stress occurring during barley's reproductive development. Except for the first sowing date at WH in 2018 (WH18NS), the trials were supplementarily irrigated during grain filling to reduce the confounding effect of drought.

**Table 1:** Summary of sowing dates, awn emergence times, and weather experienced in trials at Wongan Hills (WH) and Muresk (MK) in 2018 and 2019 crop growing seasons.

**Location**	**Wongan hills**						**Muresk**	
GPS location	30° 50' S, 116° 45' E						31° 45' S, 116° 40' E	
Soil description	Grey sandy duplex						Brown loamy earth	
Year	2018			2019			2018	2019
Annual precipitation (mm)	414			246			383	270
Environment	WH18NS	WH18LS	WH18VLS	WH19NS^b^	WH19LS	WH19VLS	MK18LS	MK19LS
Sowing date description	Normal	Late	Very late	Normal	Late	Very late	Late	Late
Sowing date	15-May	5-Jul	31-Jul	16-May	8-Jul	5-Aug	28-Jun	11-Jul
GDD from sowing to Z49 (awn emergence)	1,325	1,013	894	1,214	962	894	1,018	947
Days from sowing to Z49	105	84	69	95	72	61	89	77
Ave. daily max. temp., sowing to Z49 (°C)	18.6	18.8	20.2	19.0	20.3	22.5	18.0	20.2
Ave. daily max. temp., Z49 to physiological maturity (°C)	22.7	26.4	28.0	24.0	27.3	29.2	26.5	27.1
Days ≥30°C, sowing to Z49	0.0	1.8	2.7	0.0	3.9	7.0	0.0	2.4
Days ≥30°C, Z49 to physiological maturity	4.1	9.0	12.9	8.1	11.2	18.2	9.7	10.5
Days ≥30°C, booting/flowering^a^	0.0	1.8	2.3	0.0	3.5	3.5	0.0	1.1
Days ≥30°C, early grain filling^a^	1.1	4.6	6.9	2.6	4.6	7.5	4.2	5.1
Irrigation during grain filling (mm)	0	40	40	60	75	75	30	75

a *Based on averages for all genotypes evaluated, with “booting/flowering” defined as the period 10 days before- to 4 days after-awn emergence and “early grain-filling” from 5 to 25 days after awn emergence, respectively*.

b *Irrigated with 40 mm at sowing due to the dry season start*.

The randomised complete block designs (RCBD) were applied for all field experiments and were generated using the experimental design tool DiGGer in R (Coombes, 2018). Each plot was 10 m long, with seed sown over seven rows (22 cm row spacing). Trimming before harvest reduced the plot length to ~8 m. At MK in 2018 (MK18LS), each plot was 5 m long, trimmed to 3.5 m before harvest, with the seed sown over four rows (20 cm row spacing). The seeder was a breeding seeder with discs. MK18LS was planted as paired plots due to the shorter plot length. The left plot was used for destructive measurements and the right for grain yield and quality. The seed was sown at 2 to 4 cm depth, targeting a plant density of 150 plants m^−2^ by adjusting for kernel weight. Plots were fertilised by drilling a compound fertiliser below the seed and topdressing another compound fertiliser in front of the seeder to supply 30 kg N/ha, 28 kg P/ha, and 40.5 kg K/ha. Eight weeks after sowing, a further 40 kg N/ha was applied as a foliar fertiliser. Treating the seed and fertiliser with fungicide suppressed early disease infection, with no in-crop fungicide required. Glyphosate controlled early emerging weeds before crop establishment. In-crop weeds and diseases were controlled as needed using products registered for barley in Western Australia.

### Weather Data

Temperature data loggers (Tinytag, Hastings, UK) installed at 90 cm above the ground recorded air temperature in a bare area near each trial. Daily minimum and maximum temperatures were used to calculate the thermal time (GDD; at a base temperature of 0°C). Rainfall data (mm) was obtained from the nearest Department of Primary Industries and Regional Development (DPIRD) weather station (https://weather.agric.wa.gov.au/).

### Trait Evaluation

A total of 29 traits were measured (Supplementary Table S2) as described below.

Flowering time (DT49) was defined for each plot as the days from sowing to when 50% of plants exhibited 1 cm of awn emergence above the flag leaf (Z49) (Zadoks et al., 1974; Alqudah and Schnurbusch, 2017). Z49 is an equivalent for flowering time in barley (Alqudah and Schnurbusch, 2017). At Z49, at least 65 tillers with 1 cm of awn protruding from the boot were tagged per plot, except WH18NS, where 55 primary tillers were tagged on average. Five tagged spikes from each plot were collected at 2–3-day intervals from 7 to 14 days after flowering. and then every week to shortly after physiological maturity, except in WH18NS, where four spikes were sampled on average. The collected spikes were oven-dried for 5 days at 65°C, and the four middle grains of each spike were removed and weighed. Single grain weight (SGW, mg) was calculated by dividing the total weight of the removed grains by their number.

Grain growth characteristics were estimated by fitting a logistic function to the SGW data collected over time (Equation 1) (Zahedi and Jenner, 2003), where SGW(t) is SGW at thermal time t (GDD) after flowering, the Theoretical Final SGW at maturity (mg), s is the slope parameter that controls the steepness of the curve, TIP (GDD) is the thermal time from flowering to the inflection point (TIP), the inflection point is the point of maximum grain growth, and e is Napier's number (a mathematical constant of ~2.71828).


(1) $$\begin{array}{l} SGW\left(t\right)=\frac{Theoretical\;Final\;SGW}{{1+e}^{\left(-s\left(t-TIP\right)\right)}} \end{array}$$

The GFR reaches maximum at the inflection point when *SGW*(t) = 0.5 × (*Theoretical Final SGW*), so the maximum GRF (GFRmax, mg GDD^−1^) can be calculated using the first derivative of the logistic curve (Equation 2):


(2) $$\begin{array}{l} GFR_{\max}={\frac{d\left(SGW\left(t\right)\right)}{d\left(t\right)}|}_{SGW\left(t\right)=0.5\cdot \left(\text{Theoretical Final }SGW\right)}\\ ={\frac{s\cdot SGW\left(t\right)\cdot \left(\left(\text{Theoretical Final }SGW\right)-SGW\left(t\right)\right)}{\left(\text{Theoretical Final }SGW\right)}|}_{SGW\left(t\right)=0.5\cdot \left(\text{Theoretical Final }SGW\right)}\\ =\frac{s\cdot \left(\text{Theoretical Final }SGW\right)}{4} \end{array}$$

The grain-filling duration (GFD, in GDD) was considered to be the period until *SGW*(*t*) = 0.95 × *Theoretical Final SGW*. Thus, GFD can be calculated by using (Equation 3):


(3) $$\begin{array}{l} GFD=\frac{s.TIP+2.944}{s} \end{array}$$

The average grain-filling rate (GFR; mg GDD^−1^) was calculated using Equation 4 (Wang et al., 2009).


(4) $$\begin{array}{l} GFR=\frac{Theoretical\;Final\;SGW}{GFD} \end{array}$$

Tagged tillers were collected at 7, 14, and 49 days after Z49, except in WH19NS, in which stem samples were also collected at 21 days after Z49 in addition to 7 and 14 days after Z49, with the spikes used in the grain growth study. At each sampling time, the tillers were cut at the soil surface, and the leaf blades, at the auricle, and the spike, at the junction of the peduncle and head, were removed. The stem samples (whole stem and peduncle) were then oven-dried for 5 days at 65°C. Each dried sample was weighed, chopped into 5 mm segments, placed in a cyclone twister mill (ZM200, Retsch Co., Germany), and reduced to a fine powder. The ground samples (200 mg) were transferred into 125 ml Erlenmeyer flasks, to which 30 ml of deionized water were added. For 1 h, the flasks were sealed and placed in a shaking hot water bath (90°C; 100 RPM). After cooling, the samples were filtered into tubes, diluted to 50 ml, and refrigerated at −20°C until analysis. The WSC of each sample was quantified using the anthrone method (Yemm and Willis, 1954), using absorbance at 620 nm on a UV-visible light spectrophotometer (Model UV-120, MIOSTECH, USA) and fructose as the standard.

Water-soluble carbohydrate content (mg) was calculated by multiplying WSC concentration (mg g^−1^) by stem dry weight (g). Maximum WSC (WSCmax, mg) was defined as the highest WSC content from the samples collected at either 7, 14, or 21 days after Z49, and the minimum WSC (WSCmin, mg) was defined as the WSC content at 49 days after Z49. The amount of mobilised WSC (MWSC, mg) was calculated as the difference between the WSCmax and WSCmin. WSC mobilisation efficiency (WSCME, %) was calculated as the fraction of the maximum WSC content mobilised (Equation 5).


(5) $$\begin{array}{l} WSCME=\frac{MWSC}{WSCmax}\;\times 100 \end{array}$$

Mean relative chlorophyll content of the penultimate leaves of three to five tagged primary tillers per plot was measured using a portable SPAD chlorophyll metre (SPAD-502, Minolta Co. Ltd., Japan) weekly starting at 14 days after Z49 and concluding at 42 days after Z49. Measurements were taken from the same tagged plants in each plot over time. The SPAD values were normalised to the SPAD value at 14 days after Z49 and the area under the curve of the normalised SPAD values (NAUSC) during grain-filling (Equation 6).


(6) $$\begin{array}{l} NAUSC=\overset{n-1}{\underset{i=1}{\sum }}\left[\left(\frac{x_{i}+x_{i+1}}{2}\right)\times \left(t_{i+1}-t_{i}\right)\right] \end{array}$$

*x*_*i*_ is the relative chlorophyll content (normalised SPAD units) on the *i*^*th*^ date, *t*_*i*_ is the corresponding thermal time after flowering of the date on which the chlorophyll content was measured, and *n* is the number of dates on which chlorophyll content was recorded.

The penultimate leaf length and width were measured as the length of the blade, and width was recorded at the widest point using a ruler at ~20 days after Z49 on five randomly selected tagged tillers per plot.

Each plot's duration to physiological maturity was the period from sowing to when 75% of the plants exhibited 95% spike and peduncle senescence (DTM). At physiological maturity, before harvest, five tagged tillers were randomly selected to estimate the following traits after being oven-dried for 5 days at 65°C. Head length was measured from the peduncle collar to the tip of the spike, excluding awns (HL, cm) using a ruler. Grain number spike^−1^ (GNS) counts fertile grains per spike, while sterile kernels are reflected in the sterile floret number per spike (SFNS). After removing all the grains, grain weight per spike (GWS, g) was calculated. The harvest index (HI, %) was calculated as (GWS/the primary tiller above ground biomass) × 100. Stem length (SL, cm) was measured from five randomly selected tagged tillers per plot from the soil surface to below the collar using a metre ruler.

Plots were harvested at maturity with an experimental harvester when the grain moisture was at ~11% moisture, with a subsample collected (~1 kg). Grain yield data was collected for each genotype in all environments (except WH18NS). The influence of heat stress on grain yield will be presented in a separate paper. This paper focuses on the implication of heat stress on physical grain quality traits like grain plumpness, which are vital in managing future climate change risk for malting and brewing end-use. The grain subsample was de-awned, cleaned over a 1.5 mm slotted screen (Pfeuffer Sample Cleaner Model SLN3, Pfeuffer GmbH, Germany), and used for the physical grain quality measurements. Test weight (TW, kg/hl) was determined by a chondrometer equipped with a 500 ml cylinder (or 210 ml cylinder if there was not enough sample to fill the 500 ml cylinder). Kernel weight (TGW, g) from a thousand-grain sample (Pfeuffer Contador V1 seed counter, Pfeuffer GmbH, Germany) was calculated after oven drying for 5 days at 65°C. Grain length (GLe, mm), width (GWi, mm), and thickness (GTh, mm) were measured on a 300- to 400-grain sample using a digital image analyser (SeedCount SC6000R, Next Instruments, NSW). A 100 g sample from each plot was graded on a screening machine (Pfeuffer 4K Sortimat, Pfeuffer GmbH, Germany) with a 2.2, 2.5, 2.8, and 3.1 mm screen stack for 2 min. Screening's (Scr, %) data are presented as percent of grain passing through the 2.2 mm screen, while retention (Ret, %) is the percent retained on a 2.5 mm screen (Ret), and grain plumpness (GP, %) is the percent retained on a 2.8 mm screen. Grain protein percent (GPr, %) was predicted by near-infrared (NIR) analysis (FOSS XDS, FOSS NIR Systems Inc., USA) using calibrations developed by DPIRD in partnership with the Australian Export Grains Innovation Centre (AEGIC).

### Estimation of Heat Tolerance Index (HTI)

To mitigate differences in crop phenology (heat escape) and trait potential (i.e., the trait value under normal growing conditions), and to calculate the heat tolerance indices for TGW, GP, and Ret, the multiple regression approach by Bidinger et al. (1987) was employed. This approach has shown that the residual trait value after removing the effects of heat escape and the trait potential of a genotype gives a good indication of the heat response of that genotype. The approach considers the trait value under stress conditions (Ŷ_*s*_) as a function of the trait potential (i.e., the trait value under NS condition, *Y*_*p*_), time to flowering under stress condition (i.e., delayed sown condition, *F*_*s*_), and a stress tolerance index (Equation 7).


(7) $$\begin{array}{l} {Ŷ}_{s}=a+b.Y_{p}+c.F_{s}+HTI+E \end{array}$$

*E* is the random error with zero mean and the variances σ, *b*, and *c* are regression coefficients, while *a* is the intercept.

The heat tolerance index (HTI) is then calculated (Equation 8). *Y*_*s*_ is the actual trait value under heat stress conditions, Ŷ_*s*_ is the estimated trait value under heat stress by the multiple regression model, and *S.E*. indicates the standard error of the estimated trait value.


(8) $$\begin{array}{l} HTI=\;\frac{Y_{s}-{Ŷ}_{s}}{S.E\left({Ŷ}_{s}\right)} \end{array}$$

WH18NS and WH19NS were chosen as the baseline for calculating HTI for each WH and MK late or very late sown environment. In each growing season, they were exposed to the lowest average temperature and least number of days above 30°C. An assumption made in calculating the HTI at MK was that the main difference between the WH and MK environments was air temperature. Both sites were irrigated fortnightly during grain filling with an overhead boom irrigator (Supplementary Figure S1) supplying 15–20 mm unless there was precipitation at or around that target rate. The purpose of the irrigation was to reduce the confounding effect of drought, but there may have been further factors that influenced the HTI.

### Statistical Analysis

Each year-by-location-by-sowing date combination was considered as a separate environment, giving a total of eight environments. Linear mixed models were fitted with ASReml-R (version 4.1.0) (Butler et al., 2018) in the analyses of the evaluated traits in each environment, where the variance parameters in the mixed models were estimated using the residual maximum likelihood (REML) procedure of Patterson and Thompson (1971). For each trait in each environment, spatial variations were examined, including local autocorrelations, global trends, and extraneous variations. The blocking structures of the experiments were fitted as random effects. Spatial trends and residual variances with auto-regressive correlation at first-order for rows and columns were examined and fitted when the global trends and autocorrelations were significant. Likelihood ratio tests were used for random effects, and conditional Wald tests (Kenward and Roger, 1997) were used for fixed effects. Residual diagnostics were performed to examine the validity of the model assumption of normality and homogeneity of variance. For each fitted model, the empirical best unbiased linear estimates (eBLUEs) were produced. Broad sense heritability (*H*^2^) was estimated for each trait across all environments (Equation 9), where *r* is the number of replicates, *e* is the number of environments, σ^2^ is error variance, $\sigma_{g}^{2}$ is genotypic variance, and $\sigma_{ge}^{2}$ is genotype by environment interaction variance.


(9) $$\begin{array}{l} H^{2}=\sigma_{g}^{2}\;/\left[\sigma_{g\;}^{2}+\left(\sigma_{ge}^{2}/\text{e}\right)+\left(\sigma^{2}/\text{r.e}\right)\right] \end{array}$$

The static stability index was calculated according to environmental variance (*S*^2^) (Roemer, 1917) (Equation 10).


(10) $$\begin{array}{l} S^{2}{}_{xi}=\frac{{\sum \left(X_{ij}-{\bar{X}}_{i.}\right)}^{2}}{\left(E-1\right)} \end{array}$$

In the static stability index, *X*_*ij*_ is the observed trait value of the genotype *i* in the environment *j*, ${\bar{X}}_{i.}$ is the average trait value of the genotype *i* across environments, and *E* is the number of environments. The dynamic stability index was calculated according to Wricke's ecovalence (*W*^2^) (Wricke, 1962) (Equation 11).


(11) $$\begin{array}{l} {W^{2}}_{i}={\sum \left(X_{ij}-{\bar{X}}_{i.}-\;{\bar{X}}_{.j}+{\bar{X}}_{..}\right)}^{2} \end{array}$$

In the dynamic stability index, *X*_*ij*_ is the observed trait value of the genotype *i* in the environment *j*; ${\bar{X}}_{i.}$ is the average trait value of the genotype *i* across treatments; ${\bar{X}}_{.j}\;$is the average trait value across environment *j* of all genotypes; and ${\bar{X}}_{..}$ is the grand mean and average of all ${\bar{X}}_{.j}$. Hence, *W*^2^ states the stability dependent on the pool of genotypes evaluated by taking averages of all genotypes (${\bar{X}}_{i.}\;$ and ${\bar{X}}_{..}$) into account. At the same time, *S*^2^ is a function of only the specific genotype in question.

Pearson correlation coefficients and stepwise multiple regression analyses studied the relationship between the primary traits of interest and the traits measured under normal or delayed conditions, and between HTIs and traits measured under delayed sown conditions. Principal component analyses (PCA) were also conducted and provided as Supplementary Materials.

## Results

### Exposure of Genotypes to Heat Stress

**Figure 1:**
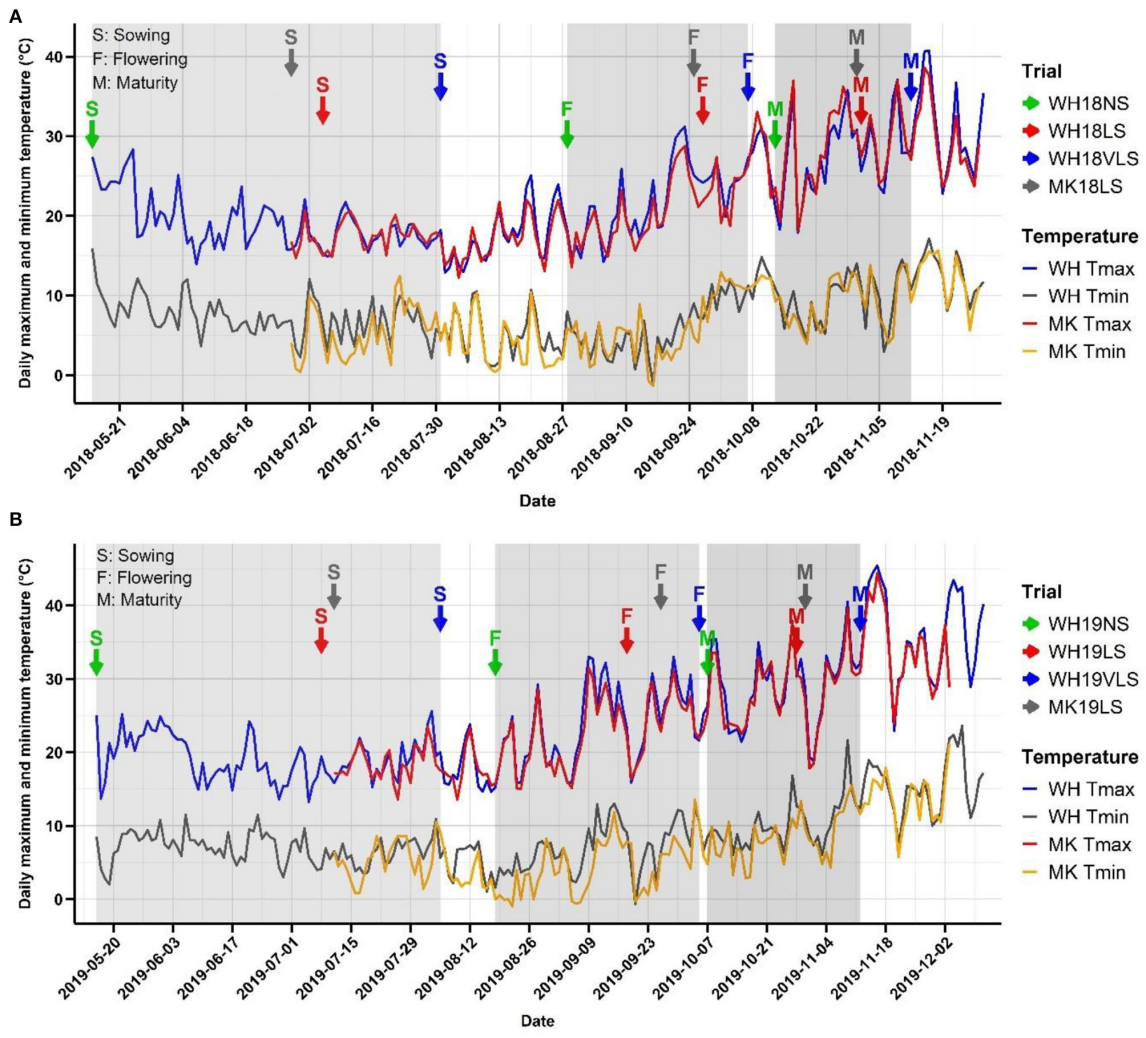
Minimum and maximum temperatures experienced in each trial at Wongan Hills (WH) and Muresk (MK) in **(A)** 2018 and **(B)** 2019 crop growing seasons. The time series line plots in blue and grey, and red and orange indicate the daily maximum and minimum temperature at WH and MK, respectively. The arrows in green, red, and blue colours indicate normal (NS), late (LS), and very late sown (VLS) trials at WH, respectively, and the grey arrows indicate LS trials at MK. S, F, and M indicate sowing, average flowering, and physiological maturity dates in each trial, respectively.

Across both growing seasons, air temperatures were milder during the vegetative stage (before Z49) than during flowering and grain filling (after Z49) (Table 1 and Figure 1). The 2019 growing season was hotter with more heat events (days > 30°C) at early to mid-grain-filling, lower total precipitation, and a generally shorter growing season than 2018. Z49 occurred in mid- to late-August in NS trials and mid-September to early-October in delayed sown trials, depending on the growing season, location, and sowing time (Figure 1). Exposure to higher daily maximum temperatures and a higher number of heat events, especially during the booting and grain-filling stages of development, was seen in delayed sown plots. At WH on average, VLS barley was exposed to 15 days above 30°C after Z49, compared to 10 days for LS and 6 days for NS barley. There were, on average, 7 days above 30°C for VLS barley during early grain filling compared to 5 for LS and 2 for NS barley. In some of the trials, a few frost events (−1.3 to 2.0°C) occurred during the booting and early grain-filling development stages (Figure 1). However, it is unlikely that those frost events significantly impacted plant performance, as crop canopy air temperatures of −3.5 to −4.5°C and below are required to damage barley when it is at its sensitive reproductive stage (Frederiks et al., 2015).

### Variance Components, Broad-Sense Heritability, and the Association of Each Trait Across Environments

The analysis of variance for genotype (G), environment (E), and G × E effects were highly significant (Table 2). Variation (%) attributed to G was more significant in 50% of the traits than that attributed to E or the interaction between G × E. The E effects were, however, more prominent than G or G × E effects for 7 of the 29 traits (DT49, GNS, SFNS, GFD, MWSC, LL, and LW), while the impact of G × E was greater than those of G or E effects for four traits (GFRmax, WSCmin, WSCME, and NAUSC). There was also a similar variation for three traits (GWS, TIP, and WSCmax).

**Table 2:** Percent of variance attributable to genetic (G), environment (E), G × E, and other effects, and broad-sense heritability estimates (*H*^2^).

**Trait**	**Variance component^¥^**				
	**G**	**E**	**G × E**	**Residual**	***H^**2**^***
DT49 (days)	33.11^***^	55.66^***^	9.70^***^	1.53	0.96
DTM (days)	43.51^***^	30.75^***^	20.12^***^	5.62	0.93
TW (kg/hL)	62.10^***^	2.54^***^	25.01^***^	10.35	0.94
TGW (g)	74.71^***^	1.69^***^	18.45^***^	5.15	0.96
GP (%)	68.66^***^	8.99^***^	19.05^***^	3.30	0.96
Ret (%)	45.67^***^	12.78^***^	35.70^***^	5.85	0.88
Scr (%)	41.50^***^	23.96^***^	24.56^***^	9.98	0.91
GLe (mm)	88.74^***^	0.67^***^	6.54^***^	4.05	0.99
GWi (mm)	57.46^***^	11.93^***^	22.51^***^	8.10	0.94
GTh (mm)	61.18^***^	4.16^***^	24.89^***^	9.77	0.94
GWS (g)	26.00^***^	26.78^***^	30.34^***^	16.88	0.83
GNS	28.05^***^	50.38^***^	13.56^***^	8.01	0.93
SFNS	13.03^***^	39.76^***^	25.96^***^	21.25	0.70
SGW (mg)^†^	57.07^***^	8.65^***^	24.26^***^	10.02	0.93
GFR (mg GDD^−1^)^†^	43.82^***^	11.51^***^	28.08^***^	16.59	0.89
GFRmax (mg GDD^−1^)^†^	29.12^***^	8.41^***^	36.40^***^	26.07	0.78
GFD (GDD)^†^	16.85^***^	39.23^***^	24.76^***^	19.16	0.75
TIP (GDD)^†^	28.01^***^	27.86^***^	26.94^***^	17.19	0.83
WSCmax (mg)^†^	28.85^***^	29.52^***^	26.77^***^	14.86	0.84
WSCmin (mg)^†^	23.32^***^	9.00^***^	40.01^***^	27.67	0.70
MWSC (mg)^†^	22.81^***^	32.46^***^	27.33^***^	17.40	0.79
WSCME (%)^†^	16.04^***^	3.60^***^	51.25^***^	29.11	0.45
NAUSC^†^	22.95^***^	18.65^***^	35.08^***^	23.32	0.73
HL (cm)	65.89^***^	2.21^***^	20.33^***^	11.57	0.95
SL (cm)	48.68^***^	10.04^***^	30.66^***^	10.62	0.91
LL (cm)^†^	16.88^***^	59.79^***^	15.04^***^	8.29	0.85
LW (cm)^†^	19.32^***^	57.88^***^	12.84^***^	9.96	0.88
HI (%)	36.25^***^	10.14^***^	35.10^***^	18.51	0.86
GPr (%)	38.49^***^	25.18^***^	23.75^***^	12.58	0.91

*DT49, days from sowing to awn emergence; DTM, days from sowing to physiological maturity; TW, test weight; TGW, thousand-grain weight; GP, grain plumpness (% grains >2.8 mm); Ret, retention (% grains >2.5 mm); Scr, screenings (% grains <2.2 mm), GLe, grain length; GWi, grain width; GTh, grain thickness; GWS, grain weight spike^−1^; GNS, grain number spike^−1^; SFNS, sterile floret number spike^−1^; SGW, single grain weight; GFR, grain-filling rate; GFRmax, maximum grain-filling rate; GFD, grain-filling duration; TIP, thermal time from awn emergence to grain growth inflection point; WSCmax, maximum stem water-soluble carbohydrate (WSC) content; WSCmin, minimum stem WSC content; MWSC, mobilised WSC during grain-filling; WSCME, WSC mobilisation efficiency; NAUSC, normalised area under SPAD curve; HL, head length; SL, stem length; LL, penultimate leaf length; LW, penultimate leaf width; HI, harvest index; GPr, grain protein percentage*.

¥ *Variance component of each effect divided by the total of all variance components, genotype (G), environment (E), G × E and residual*.

† *Measured in seven environments except WH18VLS*.

*H^2^ is broad-sense heritability*.

*** *Showing significance level at p < 0.001*.

Broad-sense heritability estimates were high (range, 0.70 to 0.99), except for WSCM which had a moderate heritability (0.45) (Table 2). Heritability was very strong (above 0.90) for DT49, DTM, grain weight, and size parameters (TW, TGW, GP, Scr, GLe, GWi, GTh, and SGW), HL, SL and GPr. GFR had the highest heritability (0.89), followed by WSCmax and TIP (0.84 and 0.83, respectively) among the physiological traits.

The correlations of trait values across environments were computed using Pearson's correlation coefficients (Supplementary Figure S2). The traits values were significantly positively correlated across environments for all traits in most cases, reflecting the strong genetic effect and high heritability observed for the traits. The exceptions being WSCmin, WSCME, and NAUSC, which were less correlated across environments, reflecting a lower heritability and/or a strong G × E effect for these traits.

### Overall Environmental Effects

**Figure 2:**
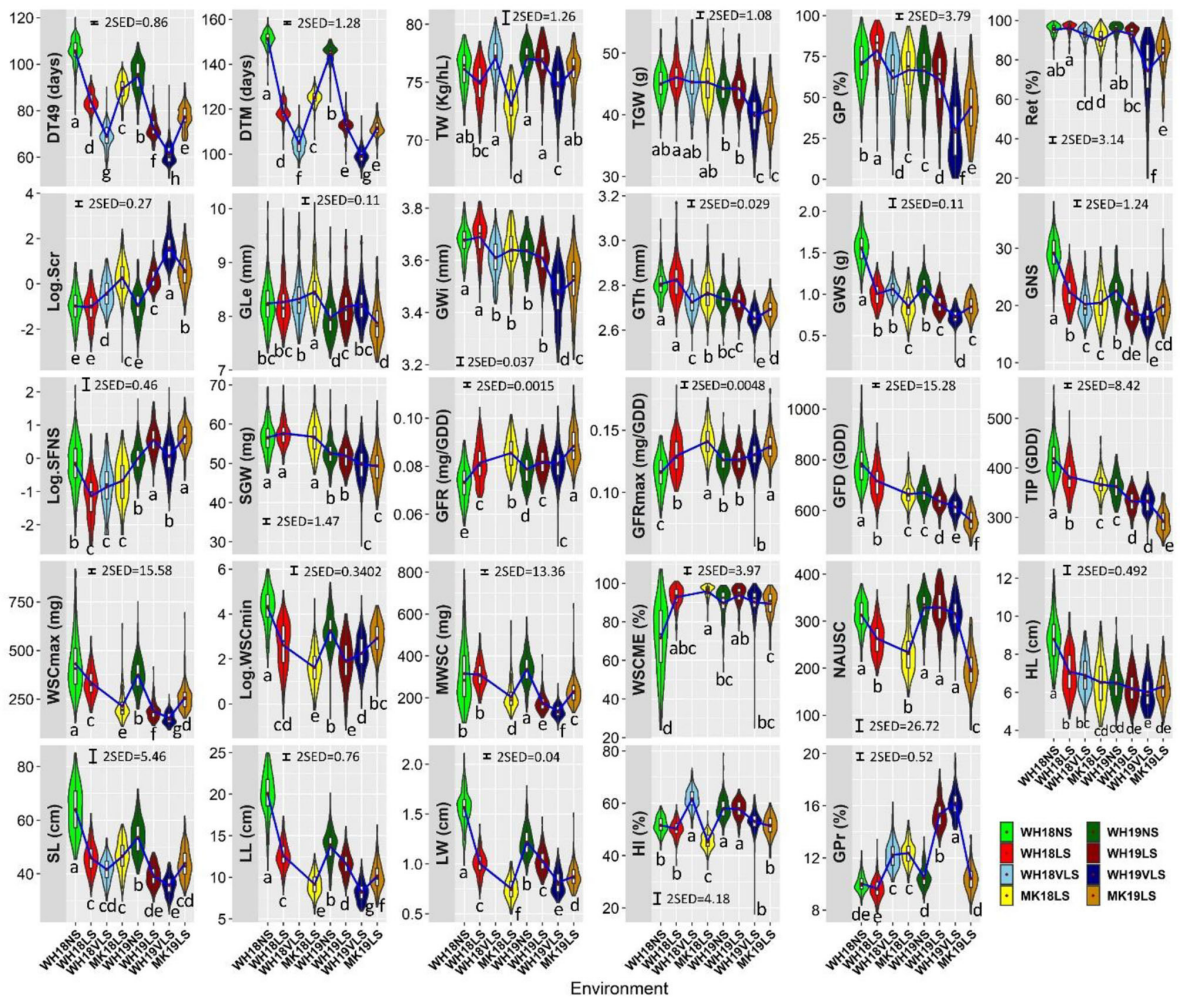
Trait distributions in WH18NS (light green), WH18LS (light red), WH18VLS (sky blue), MK18LS (yellow), WH19NS (dark green), WH19LS (dark red), WH19VLS (navy blue), and MK19LS (orange) environments. The length of the boxes show the interquartile range, the horizontal line within the box indicating the median, red diamonds within the box representing the mean, the whiskers the variability within 1.5 interquartile range of the upper and lower quartiles, and the widths of the violin plots indicate the probability density of the data at different values. The blue lines connect the means of each environment for each trait. Environment and trait definitions are as defined for Tables 1, 2, respectively. Bars represent 2 × standard error of differences (SED) with α = 0.05. Different letters below the violin plots represent significant differences between environments for each trait at significance level of 0.05.

The expression of each trait varied with growing environment and genotype (Figure 2 and Supplementary Figure S3), with environmental conditions during 2018 generally more favourable than those in 2019. The delayed sown effects were smaller in 2018 than in 2019, while VLS had a larger response than LS at WH in both seasons. The larger responses observed with VLS at WH in 2019 are consistent with higher levels of heat stress.

Delayed sowing plants generally had the following characteristics: shorter durations of their developmental phases (DT49, DTM, GFD, and TIP), reduced primary tiller fertility, shorter plants with smaller leaves (width and length), smaller and lighter heads with fewer grains per head of smaller grain size, lower maximum and minimum WSC, lower MWSC during grain-filling, shorter green leaf area duration, lower HI; higher WSCME, and faster GFR (Figure 2). Overall, delayed sowing increased the risk of delivering feed grade barley due to lower grain plumpness, higher screenings, and higher grain protein. The effect on test weight was generally small (and often insignificant).

On average, delayed sowing reduced DT49 and DTM in 16–36 days and 26–46 days, respectively. The reduction in DT49 and DTM relative to NS were similar across the two seasons and were largest for VLS, with higher average temperatures and longer days during the entire growing period.

Spike sterility behaved differently in the two seasons due to delayed sowing. Particularly, lower in 2018 and higher in 2019. Delayed sowing considerably affected the grain growth components. On average, relative to NS, delayed sowing significantly increased GFR and GFRmax (3.1–21.4%, except GFRmax in WH19LS and WH19VLS), whereas significantly reduced GFD and the related trait TIP (5.1–19.0%).

Single grain weight behaved differently in the two seasons. Generally, the GFR was increased larger than the concomitant decrease in grain-filling duration at delayed sown environments relative to NS in 2018, while the reverse happened in 2019. In line with this, relative to NS, delayed sowing slightly increased SGW in 2018, whereas it tended to reduce SGW in 2019 (by 1.3–6.2%; the effect was insignificant in WH19LS).

Stem WSC content was generally much higher at early grain-filling (~375.0–431.0 mg under NS and ~153.0–336.0 mg under delayed sown) than maturity (~26.0–72.0 mg under NS and ~5.0–19.0 mg under delayed sowing). Reduced stem volume and less WSC deposition (probably due to reduced photosynthesis; data not shown) were associated with the decline in WSCmax by 22.2–59.3%. In line with this, MWSC was lower by 31.3–59.1% in delayed sown environments relative to NS (excluding WH18LS which had only slightly lower MWSC relative to NS). Stem WSCmin at maturity were also lower by 28.3–92.9%, with delayed sowing indicating higher exhaustion of WSC. Nevertheless, WSCME was only significantly increased in response to delayed sowing in 2018 (29.2–33.5) and only showed an insignificant increase in 2019 (except in MK19LS, which had slightly lower WSCME relative to NS).

Generally, delayed sowing accelerated senescence and reduced normalised area under the penultimate leaf's SPAD decline curve (NAUSC). NAUSC was significantly decreased in WH18LS, MK18LS, and MK19LS relative to the respective NS (16.0–40.1%), while it showed an insignificant decline in WH19VLS and a negligible increase in WH19LS relative to the respective NS.

The general reduction in HI with delayed sowing was only significant in MK18LS, WH19VLS, and MK19LS (11.8, 8.8, and 11.7%, respectively), except for WH18VLS which showed 19.7% higher HI relative to respective NS.

### Physical Grain Quality Heat Tolerance Index (HTI)

Thousand grain weight (TGW), GP, and Ret values in delayed sown conditions were significantly correlated with their potentials (i.e., the trait *per se* value under NS conditions) and DT49 (Supplementary Table S3). Therefore, a considerable variation in physical grain quality among the genotypes due to delayed sowing could be attributed to variation in their trait potential (genetic weight and size) and DT49 (heat escape). Due to these confounding factors, HTIs were calculated for TGW, GP, and Ret using linear terms for both the trait potentials and time to flowering as described by Bidinger et al. (1987). In calculating HTIs, its distribution is symmetric with a mean of 0.

Genotypes varied substantially for the physical grain quality heat responses and their heat response stability (Supplementary Table S4). TGW, GP, and Ret HTIs (TGW.HTI, GP.HTI, and Ret.HTI, respectively) ranged from 1.99 to −2.30, 1.72 to −2.33, and 1.86 to −2.35, respectively (Supplementary Table S4). TGW.HTI, GP.HTI, and Ret.HTI was moderate to strongly correlated across environments (Supplementary Table S5), suggesting that HTI is a universal response index to heat stress.

There was good correspondence between genotypes that responded the least or the most to delayed sowing for TGW.HTI, GP.HTI, and Ret.HTI. For TGW.HTI, Capstan, Fathom, Lockyer, Sloop VIC, and VB0916 were the best, while Baudin-Hs 2, Dampier, Tallon, and Yan 95168 were the worst-performing. For GP, Lockyer, Cowabbie, and 08S917N-226 were the best, while Fitzgerald, Tallon and Yan 95168 were the worst-performing. For Ret, Capstan, Lockyer, and VB0916 were the best, while Tallon, WA8964, and Yan 95168 were the worst-performing. The heat-tolerant genotypes tended to have more stability. In contrast, the susceptible genotypes appeared to be more responsive to the environmental changes and consequently had less stability in their response (Supplementary Table S4).

### The Relative Contribution of Physical Grain Quality Potentials, Time to Flowering, and Heat Tolerance to Physical Grain Quality Performance Under Delayed Sowing

**Figure 3:**
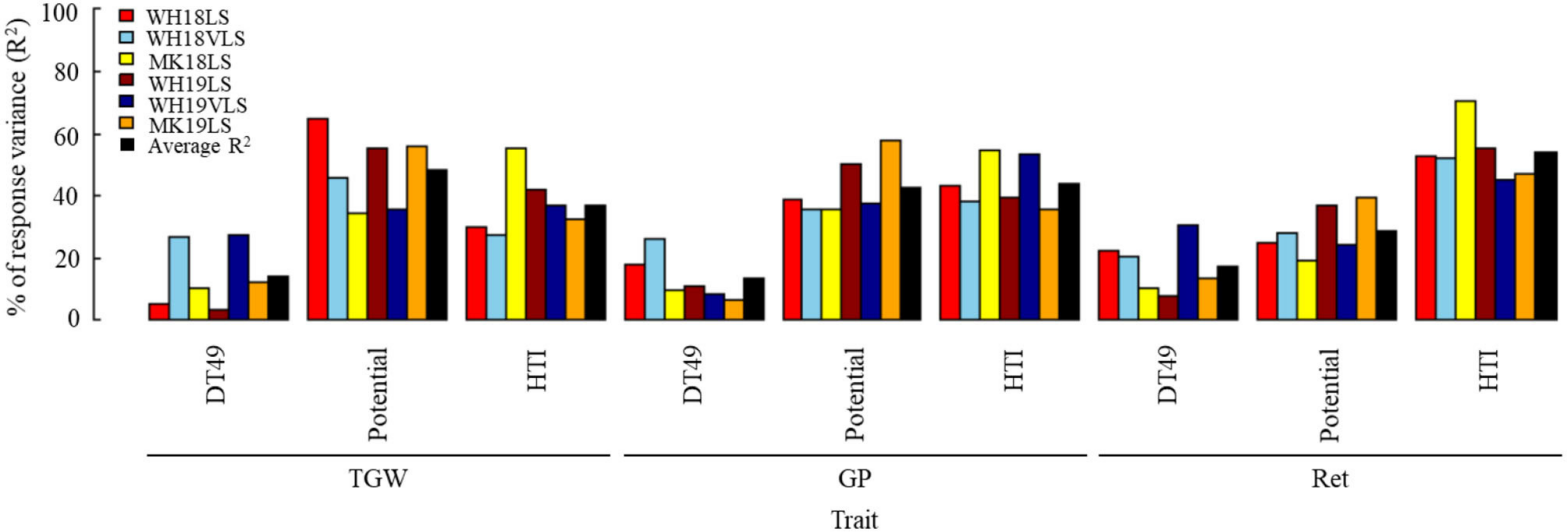
Estimated contribution (% of the variance, *R*^2^) of the trait potential, time to flowering, and heat tolerance index to thousand-grain weight (TGW), grain plumpness (GP), and retention (Ret) under delayed sown/heat-stress conditions. Environment definitions are as defined in Table 1.

The HTI explained 37.2, 44.1, and 53.4% of the variation in TGW, GP, and Ret, respectively (Figure 3 and Supplementary Table S6). On average, TGW, GP, and Ret potentials explained ~48.6, 42.7, and 28.7% of the variations in TGW, GP, and Ret, while DT49 made a lower contribution to physical grain quality. DT49 accounted for 14.2, 13.2, and 17.5% of the observed variation in TGW, GP, and Ret under delayed sown, respectively.

Heat tolerance index appeared to be the primary factor determining Ret, while TGW potential was the main determinant of TGW, and GP potential and HTI were equally crucial in determining GP. The combined factors accounted for 100% of the observed variability for the physical grain quality parameters. This suggests that this analysis method effectively estimates major determinants of physical grain quality under delayed sown/heat-stressed growing conditions.

### Association of Selected Physical Grain Quality Parameters With the Traits Evaluated Within Environments

Thousand grain weight (TGW), GP, and Ret were negatively correlated with DT49 and DTM in pairwise correlations across delayed sown environments in both seasons (Table 3 and Supplementary Table S7). The negative correlations between phenology (DT49 and DTM) and the physical grain quality parameters were either missing or weaker in NS environments than delayed sown environments. Genotypes with heavier and plumper grains tended to have earlier flowering and maturity under delayed sowing. Unsurprisingly, TGW, GP, and Ret were significantly correlated with one another and with other grain weight and size parameters (i.e., TW, Scr, GLe, GWi, GTh, SGW and GWS) across environments (Table 3). TGW, GP, and Ret showed stronger correlations with GWi and GTh than GLe in delayed sown environments, suggesting that the alteration in lateral dimension was probably more important in determining grain weight and size under heat-stress conditions. TGW, GP, and Ret were negatively correlated (moderate to relatively strong) with GNS and HL across all environments (except in WH18NS). Therefore, genotypes with heavier and plumper grains tended to have shorter heads with lower grain numbers (i.e., smaller sink size), which probably led to less competition between grains for photosynthates.

**Table 3:** Correlations between thousand-grain weight (TGW), grain plumpness (GP), and retention (Ret), and traits measured in each environment.

	**Environment**																							
	**WH18NS**			**WH18LS**			**WH18VLS**			**MK18LS**			**WH19NS**			**WH19LS**			**WH19VLS**			**MK19LS**		
**Trait**	**TGW**	**GP**	**Ret**	**TGW**	**GP**	**Ret**	**TGW**	**GP**	**Ret**	**TGW**	**GP**	**Ret**	**TGW**	**GP**	**Ret**	**TGW**	**GP**	**Ret**	**TGW**	**GP**	**Ret**	**TGW**	**GP**	**Ret**
DT49	−0.03	−0.09	−0.04	−0.25^**^	−0.46^***^	−0.48^***^	−0.53^***^	−0.54^***^	−0.46^***^	−0.34^***^	−0.31^***^	−0.31^***^	−0.20^*^	−0.28^***^	−0.29^***^	−0.24^**^	−0.43^***^	−0.38^***^	−0.60^***^	−0.38^***^	−0.64^***^	−0.42^***^	−0.34^***^	−0.46^***^
DTM	0.00	−0.07	−0.05	−0.28^***^	−0.42^***^	−0.47^***^	−0.55^***^	−0.50^***^	−0.43^***^	−0.14	−0.21^**^	−0.19^*^	−0.18^*^	−0.30^***^	−0.23^**^	−0.14	−0.32^***^	−0.25^**^	−0.49^***^	−0.28^***^	−0.52^***^	−0.33^***^	−0.34^***^	−0.46^***^
TW	−0.04	0.22^**^	0.23^**^	−0.24^**^	0.06	0.05	−0.01	0.43^***^	0.48^***^	0.24^**^	0.42^***^	0.47^***^	−0.12	0.33^***^	0.36^***^	−0.19^*^	0.19^*^	0.35^***^	0.40^***^	0.59^***^	0.70^***^	0.15	0.40^***^	0.47^***^
TGW		0.53^***^	0.48^***^		0.56^***^	0.55^***^		0.58^***^	0.46^***^		0.58^***^	0.66^***^		0.48^***^	0.47^***^		0.43^***^	0.38^***^		0.58^***^	0.75^***^		0.58^***^	0.71^***^
GP	0.53^***^		0.89^***^	0.56^***^		0.94^***^	0.58^***^		0.84^***^	0.58^***^		0.90^***^	0.48^***^		0.88^***^	0.43^***^		0.87^***^	0.58^***^		0.81^***^	0.58^***^		0.86^***^
Ret	0.48^***^	0.89^***^		0.55^***^	0.94^***^		0.46^***^	0.84^***^		0.66^***^	0.90^***^		0.47^***^	0.88^***^		0.38^***^	0.87^***^		0.75^***^	0.81^***^		0.71^***^	0.86^***^	
Scr	−0.41^***^	−0.74^***^	−0.91^***^	−0.58^***^	−0.83^***^	−0.91^***^	−0.46^***^	−0.78^***^	−0.88^***^	−0.64^***^	−0.79^***^	−0.88^***^	−0.48^***^	−0.74^***^	−0.90^***^	−0.28^***^	−0.50^***^	−0.76^***^	−0.73^***^	−0.63^***^	−0.90^***^	−0.70^***^	−0.70^***^	−0.91^***^
GLe	0.63^***^	−0.08	−0.08	0.61^***^	−0.11	−0.07	0.65^***^	−0.06	−0.15	0.46^***^	−0.16^*^	−0.04	0.60^***^	−0.26^**^	−0.22^**^	0.60^***^	−0.19^*^	−0.26^**^	0.38^***^	−0.30^***^	−0.17^*^	0.61^***^	−0.07	0.07
GWi	0.55^***^	0.68^***^	0.62^***^	0.69^***^	0.77^***^	0.76^***^	0.75^***^	0.70^***^	0.61^***^	0.72^***^	0.73^***^	0.77^***^	0.63^***^	0.78^***^	0.69^***^	0.65^***^	0.65^***^	0.59^***^	0.86^***^	0.71^***^	0.80^***^	0.85^***^	0.72^***^	0.80^***^
GTh	0.31^***^	0.64^***^	0.47^***^	0.52^***^	0.75^***^	0.68^***^	0.46^***^	0.76^***^	0.65^***^	0.51^***^	0.75^***^	0.66^***^	0.35^***^	0.82^***^	0.68^***^	0.33^***^	0.74^***^	0.62^***^	0.25^**^	0.58^***^	0.49^***^	0.28^***^	0.69^***^	0.53^***^
GWS	0.33^***^	0.17^*^	0.13	−0.16^*^	−0.25^**^	−0.21^*^	0.03	−0.07	0.00	−0.12	−0.12	−0.18^*^	0.13	−0.09	−0.12	0.02	−0.22^**^	−0.16	0.14	−0.08	0.04	0.16	0.03	0.07
GNS	−0.13	−0.01	−0.02	−0.53^***^	−0.40^***^	−0.35^***^	−0.51^***^	−0.41^***^	−0.29^***^	−0.47^***^	−0.37^***^	−0.47^***^	−0.44^***^	−0.32^***^	−0.33^***^	−0.45^***^	−0.40^***^	−0.31^***^	−0.49^***^	−0.41^***^	−0.41^***^	−0.38^***^	−0.30^***^	−0.33^***^
SFNS	−0.19^*^	−0.26^**^	−0.21^**^	−0.22^**^	−0.33^***^	−0.33^***^	−0.19^*^	−0.26^**^	−0.23^**^	−0.24^**^	−0.17^*^	−0.16^*^	−0.15	−0.21^*^	−0.23^**^	−0.13	−0.26^**^	−0.28^***^	−0.23^**^	−0.15	−0.28^***^	−0.09	−0.17^*^	−0.20^*^
SGW	0.76^***^	0.32^***^	0.27^***^	0.80^***^	0.33^***^	0.32^***^	–	–	–	0.82^***^	0.49^***^	0.58^***^	0.81^***^	0.34^***^	0.31^***^	0.82^***^	0.27^***^	0.19^*^	0.87^***^	0.48^***^	0.64^***^	0.89^***^	0.44^***^	0.58^***^
GFR	0.55^***^	0.17^*^	0.10	0.47^***^	0.06	0.05	–	–	–	0.63^***^	0.34^***^	0.41^***^	0.58^***^	0.05	0.03	0.62^***^	0.19^*^	0.07	0.70^***^	0.32^***^	0.43^***^	0.68^***^	0.29^***^	0.41^***^
GFRmax	0.46^***^	0.18^*^	0.11	0.37^***^	0.02	0.00	–	–	–	0.49^***^	0.29^***^	0.33^***^	0.51^***^	0.05	0.01	0.48^***^	0.06	−0.03	0.56^***^	0.28^***^	0.39^***^	0.56^***^	0.22^**^	0.32^***^
GFD	−0.01	0.06	0.11	0.04	0.13	0.15	–	–	–	0.21^**^	0.15	0.15	0.17^*^	0.33^***^	0.32^***^	0.21^**^	0.09	0.15	0.20^*^	0.22^**^	0.29^***^	0.37^***^	0.24^**^	0.29^***^
TIP	−0.01	0.13	0.17^*^	0.03	0.13	0.13	–	–	–	0.19^*^	0.18^*^	0.17^*^	0.10	0.30^***^	0.27^***^	0.12	−0.03	0.05	0.10	0.20^*^	0.29^***^	0.26^**^	0.19^*^	0.22^**^
WSCmax	0.10	−0.04	0.02	0.04	−0.16	−0.12	–	–	–	0.08	0.05	0.13	0.05	−0.03	−0.04	−0.03	−0.19^*^	−0.18^*^	−0.03	−0.17^*^	−0.08	−0.04	−0.13	−0.09
WSCmin	0.17^*^	0.17^*^	0.17^*^	−0.11	−0.19^*^	−0.13	–	–	–	−0.04	0.06	0.05	0.22^**^	0.23^**^	0.22^**^	−0.36^***^	−0.34^***^	−0.39^***^	−0.17^*^	−0.17^*^	−0.27^***^	−0.16^*^	0.01	−0.10
MWSC	0.02	−0.16	−0.12	0.06	−0.12	−0.11	–	–	–	0.11	0.05	0.15	−0.06	−0.15	−0.15	0.06	−0.11	−0.09	0.05	−0.09	0.06	0.01	−0.12	−0.05
WSCME	−0.11	−0.16^*^	−0.15	0.09	0.17^*^	0.09	–	–	–	0.20^*^	0.10	0.13	−0.23^**^	−0.30^***^	−0.29^***^	0.34^***^	0.31^***^	0.36^***^	0.16^*^	0.19^*^	0.31^***^	0.14	−0.04	0.09
NAUSC	0.11	0.31^***^	0.26^**^	−0.04	0.26^**^	0.19^*^	–	–	–	0.32^***^	0.12	0.15	0.14	0.30^***^	0.29^***^	0.10	0.31^***^	0.29^***^	0.16	0.31^***^	0.31^***^	0.29^***^	0.27^***^	0.30^***^
HL	−0.04	−0.06	0.00	−0.43^***^	−0.42^***^	−0.37^***^	−0.48^***^	−0.47^***^	−0.32^***^	−0.40^***^	−0.38^***^	−0.44^***^	−0.30^***^	−0.33^***^	−0.30^***^	−0.33^***^	−0.44^***^	−0.32^***^	−0.54^***^	−0.50^***^	−0.56^***^	−0.39^***^	−0.42^***^	−0.44^***^
SL	0.26^**^	0.32^***^	0.33^***^	0.13	0.11	0.16^**^	0.09	0.06	0.13	−0.05	0.14	0.06	0.25^**^	0.35^***^	0.30^***^	0.24^**^	0.25^**^	0.16^*^	−0.07	0.03	−0.03	0.31^***^	0.35^***^	0.39^***^
LL	0.38^***^	0.10	0.21^**^	0.12	0.13	0.16	–	–	–	−0.03	−0.17^*^	−0.21^**^	0.23^**^	0.04	0.10	0.18^*^	−0.04	−0.10	−0.09	−0.28^***^	−0.25^**^	0.19^*^	−0.03	0.10
LW	0.14	0.13	0.18^*^	0.01	0.01	0.01	–	–	–	0.01	−0.16^*^	−0.15	0.17^*^	−0.09	−0.02	0.02	−0.18^*^	−0.26^**^	−0.19^*^	−0.34^***^	−0.34^***^	0.13	−0.08	0.02
HI	−0.13	−0.25^**^	−0.31^***^	−0.27^***^	−0.13	−0.18^*^	0.10	0.21^**^	0.11	−0.19^*^	−0.19^*^	−0.19^*^	0.11	−0.08	−0.07	0.14	0.09	0.20^*^	0.24^**^	0.11	0.30^***^	0.13	0.01	0.09
GPr	0.26^**^	0.27^***^	0.34^***^	0.31^***^	0.34^***^	0.39^***^	0.25^**^	0.20^*^	0.08	0.20^*^	0.13	0.18^*^	0.15	0.12	0.18^*^	−0.18^*^	−0.26^**^	−0.33^***^	0.15	0.11	0.24^**^	0.01	0.00	−0.02

*Environment and trait definitions are as defined for Tables 1, 2, respectively*.

*Values are Pearson correlation coefficients and significance level indicated by*

* *p < 0.05*,

** *p < 0.01, and*

*** *p < 0.001*.

*–Indicates that the trait was not evaluated in the respective environment*.

While most physiological attributes influenced physical grain quality *per se*, grain quality was best correlated with GFR. GFR was strongly and positively correlated with SGW and TGW, with the correlation stronger with delayed sowing (Supplementary Table S7). GFR was also positively correlated (moderate to relatively strong) with GP and Ret across all delayed sown environments (except WH18LS). It only showed a weak positive correlation with GP in one NS environment (WH18NS). GFD and the related trait TIP showed weak to moderate positive correlations with TGW, GP, and Ret under normal and delayed sown conditions, mainly in the 2019 growing season. These correlations indicate that genotypes with heavier and plumper grains had higher GFRs and longer grain-filling durations. However, the GFR rate may play a relatively more important role in determining TGW, GP, and Ret (and other grain weight and size parameters) (Table 3 and Supplementary Table S7).

Normalised area under the penultimate leaf's SPAD decline curve (NAUSC) was positively correlated (moderate) with GP and Ret across all environments (except in MK18LS) and with TGW only in late sown trials at MK (i.e., MK18LS and MK19LS). Therefore, the penultimate leaf chlorophyll retention during grain-filling (stay-green) contributed to better physical grain quality under normal and delayed sown conditions. NAUSC was positively correlated with GFD and TIP across environments (Supplementary Table S7), indicating that the stay-green contributed to better physical grain quality performance via stabilisation of GFD.

The stem WSC parameters behaved differently to TGW, GP, and Ret. WSCmin and WSCME showed weak to moderate positive and negative correlations, respectively, with TGW, GP, and Ret under NS conditions, while the reverse held under delayed sown conditions. These correlations indicate that genotypes with better physical grain quality tended to have higher WSC mobilisation efficiency and lower stem WSC residuals at physiological maturity under delayed sown/heat-stress conditions. The reverse was true under NS conditions. MWSC and WSCME were positively correlated with GFR and GFRmax across environments (Supplementary Table S7), indicating a link between WSC mobilisation with better physical grain quality performance via stabilisation of GFR. WSCmax showed weak negative correlations with GP and Ret only in WH19LS and WH19VLS. This was in contrast to what was expected if higher WSC content *per se* at early grain-filling contributed to better physical grain quality under heat.

Thousand grain weight (TGW), GP, and Ret showed moderate positive correlations with SL under NS, WH19LS and MK19LS. LL was positively correlated (weak to moderate) with TGW in NS, WH19LS and MK19LS environments. It was positively correlated with Ret in WH18NS and negatively correlated with GP and Ret in MK18LS and WH19VLS. LW was positively correlated (weak) with TGW and Ret in NS and negatively correlated with TGW and grain size in some delayed sown environments, mainly WH in 2019.

Grain protein percent was generally positively correlated with the physical grain quality parameters across environments except for WH19LS, which was negatively correlated.

The relationship between the physical grain quality parameters and other traits in PCA analyses was similar to the correlation tests. The PCA indicate that most of the variation among the genotypes could be accounted for by the physical grain quality parameters and GFR under delayed sown/heat stress conditions and that they (i.e., TGW, GP, Ret, and GFR) were positively correlated to one another under such conditions (Supplementary Figure S4).

**Figure 4:**
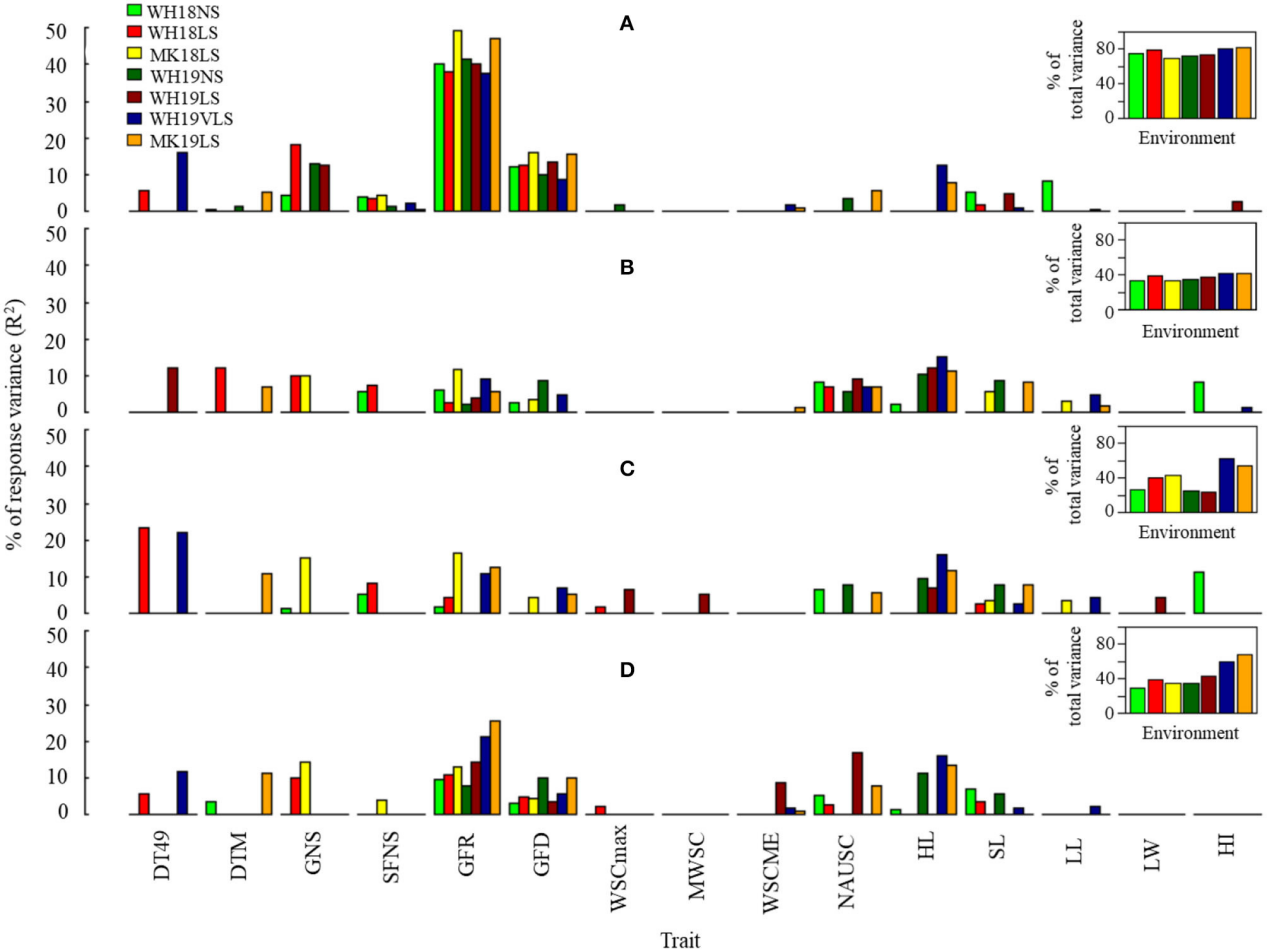
Estimated contribution (% of variance, *R*^2^) of selected phenological, agro-morphological and physiological traits to **(A)** TGW, **(B)** GP, **(C)** Ret, and **(D)** grain width (GWi) in WH18NS (light-green), WH18LS (light-red), MK18LS (yellow), WH19NS (dark-green), WH19LS (dark-red), WH19VLS (navy-blue), and MK19LS (orange) environments. Insets show the total variability explained by the best significant model in each environment (*p* < *0.0001*). Environment and trait definitions are as defined for Tables 1, 2, respectively.

The associations between TGW, GP, Ret, and GWi (as dependent variables) and selected phenological, agro-morphological, and physiological parameters (as independent variables) were tested in a stepwise multiple linear regression analysis (except for WH19VLS) (Figure 4 and Supplementary Table S8). The best model accounted for 69.7–81.8%, 32.7–41.9%, 23.1–62.8%, and 29.3–68.4% of the variation for TGW, GP, Ret, and GWi, respectively, depending on the environment (Figure 4). Grain growth components (GFR and GFD), sink size (HL and GNS), and plant stature (SL) were significant when added to the model. GP and GWi models included stay-green (NAUSC) in most environments.

Grain-filling rate was the most potent predictor for TGW and GWi across the environments. In contrast, the best predictor for GP and Ret varied across environments (generally HL and GFR in most cases). Interestingly, GFR's contribution to explaining grain size variation (GP, Ret, and GWi) was considerably stronger under delayed sown conditions in comparison with NS.

### Associations of HTIs and the Traits Evaluated Within Delayed Sown Environments

The considerable variation in agro-morphological and physiological traits' expression across delayed sown/heat-stressed environments, together with considerable variation for heat tolerance among the genotypes, provided an opportunity to test the associations of heat tolerance with other traits. This was explored to see whether the expression of specific traits was more advantageous under heat stress than others. If this were the case, selection for genotypes adapted to heat stress would be considerably simplified (Table 4).

**Table 4:** Correlations between thousand-grain weight, grain plumpness, and retention heat tolerance indices (TGW.HTI, GP.HTI, and Ret. HTI, respectively), and traits measured within each delayed sown environment.

	**Environment**																	
	**WH18LS**			**WH18VLS**			**MK18LS**			**WH19LS**			**WH19VLS**			**MK19LS**		
**Trait**	**TGW.HTI**	**GP.HTI**	**Ret.HTI**	**TGW.HTI**	**GP.HTI**	**Ret.HTI**	**TGW.HTI**	**GP.HTI**	**Ret.HTI**	**TGW.HTI**	**GP.HTI**	**Ret.HTI**	**TGW.HTI**	**GP.HTI**	**Ret.HTI**	**TGW.HTI**	**GP.HTI**	**Ret.HTI**
DT49	0.00	0.00	0.00	0.00	0.00	0.00	0.00	0.00	0.00	0.00	0.00	0.00	0.00	0.00	0.00	0.00	0.00	0.00
DTM	−0.13	−0.13	−0.12	−0.24^**^	−0.18^*^	−0.11	0.02	−0.08	−0.05	0.14	0.03	0.09	−0.06	0.04	0.00	0.01	−0.02	−0.05
TW	−0.04	0.19^*^	0.21^**^	0.08	0.36^***^	0.42^***^	0.20^*^	0.34^***^	0.41^***^	0.07	0.28^***^	0.46^***^	0.33^***^	0.32^***^	0.43^***^	0.22^**^	0.19^*^	0.32^***^
TGW	0.55^***^	0.38^***^	0.40^***^	0.52^***^	0.30^***^	0.18^*^	0.74^***^	0.50^***^	0.57^***^	0.65^***^	0.38^***^	0.30^***^	0.61^***^	0.33^***^	0.43^***^	0.57^***^	0.48^***^	0.62^***^
GP	0.34^***^	0.66^***^	0.62^***^	0.22^**^	0.62^***^	0.48^***^	0.38^***^	0.74^***^	0.68^***^	0.15	0.63^***^	0.52^***^	0.41^***^	0.73^***^	0.63^***^	0.26^**^	0.60^***^	0.56^***^
Ret	0.34^***^	0.63^***^	0.73^***^	0.11	0.43^***^	0.72^***^	0.48^***^	0.71^***^	0.84^***^	0.21^*^	0.59^***^	0.74^***^	0.46^***^	0.45^***^	0.67^***^	0.37^***^	0.48^***^	0.69^***^
Scr	−0.32^***^	−0.55^***^	−0.65^***^	−0.14	−0.42^***^	−0.58^***^	−0.48^***^	−0.62^***^	−0.73^***^	−0.37^***^	−0.47^***^	−0.77^***^	−0.44^***^	−0.26^**^	−0.53^***^	−0.38^***^	−0.38^***^	−0.61^***^
GLe	0.20^*^	−0.14	−0.12	0.28^***^	−0.15	−0.26^**^	0.22^**^	−0.11	−0.01	0.24^**^	−0.13	−0.25^**^	0.08	−0.22^**^	−0.21^*^	0.28^***^	0.15	0.21^*^
GWi	0.47^***^	0.56^***^	0.59^***^	0.40^***^	0.41^***^	0.34^***^	0.58^***^	0.61^***^	0.65^***^	0.48^***^	0.43^***^	0.42^***^	0.63^***^	0.45^***^	0.55^***^	0.49^***^	0.45^***^	0.58^***^
GTh	0.32^***^	0.38^***^	0.41^***^	0.16^*^	0.36^***^	0.33^***^	0.37^***^	0.55^***^	0.51^***^	0.18^*^	0.38^***^	0.38^***^	0.26^**^	0.37^***^	0.32^***^	0.13	0.34^***^	0.30^***^
GWS	−0.14	−0.07	0.05	−0.07	−0.10	0.05	−0.05	−0.03	−0.07	0.02	−0.16	−0.08	−0.03	−0.22^**^	−0.17^*^	0.16^*^	0.01	0.06
GNS	−0.31^***^	−0.18^*^	−0.08	−0.30^***^	−0.23^**^	−0.06	−0.24^**^	−0.20^*^	−0.30^***^	−0.23^**^	−0.29^***^	−0.16^*^	−0.30^***^	−0.35^***^	−0.34^***^	−0.11	−0.22^**^	−0.27^**^
SFNS	−0.12	−0.18^*^	−0.22^**^	−0.14	−0.20^*^	−0.15	−0.24^**^	−0.12	−0.09	0.06	−0.03	−0.12	0.01	0.08	0.08	0.01	0.06	0.04
SGW	0.34^***^	0.22^**^	0.27^**^	–	–	–	0.48^***^	0.38^***^	0.46^***^	0.43^***^	0.23^**^	0.12	0.32^***^	0.16	0.22^**^	0.44^***^	0.30^***^	0.44^***^
GFR	0.37^***^	0.27^**^	0.24^**^	–	–	–	0.45^***^	0.37^***^	0.43^***^	0.28^***^	0.26^**^	0.06	0.30^***^	0.12	0.15	0.36^***^	0.26^**^	0.42^***^
GFRmax	0.29^***^	0.22^**^	0.20^*^	–	–	–	0.35^***^	0.31^***^	0.35^***^	0.18^*^	0.12	−0.03	0.25^**^	0.11	0.14	0.28^***^	0.16	0.32^***^
GFD	−0.17^*^	−0.14	−0.08	–	–	–	0.01	−0.02	0.00	0.16^*^	−0.03	0.07	0.00	0.06	0.10	0.16	0.10	0.08
TIP	−0.20^*^	−0.14	−0.09	–	–	–	−0.01	0.01	0.04	0.06	−0.13	−0.01	−0.05	0.01	0.05	0.08	0.00	0.00
WSCmax	−0.16	−0.18^*^	−0.13	–	–	–	−0.02	0.05	0.11	−0.19^*^	−0.21^**^	−0.26^**^	−0.08	−0.19^*^	−0.03	−0.08	−0.11	−0.08
WSCmin	−0.16^*^	−0.12	−0.08	–	–	–	−0.06	0.00	0.00	−0.32^***^	−0.26^**^	−0.30^***^	0.11	0.13	0.17^*^	0.08	0.16	0.12
MWSC	−0.13	−0.16^*^	−0.13	–	–	–	0.00	0.07	0.12	−0.12	−0.16	−0.19^*^	−0.11	−0.23^**^	−0.07	−0.11	−0.14	−0.11
WSCME	0.12	0.08	0.01	–	–	–	0.07	0.06	0.08	0.23^**^	0.18^*^	0.21^**^	−0.09	−0.10	−0.07	−0.13	−0.20^*^	−0.17^*^
NAUSC	−0.14	0.00	−0.06	–	–	–	0.09	0.01	0.04	0.11	0.10	0.16^*^	−0.03	0.12	0.07	0.24^**^	0.16	0.16^*^
HL	−0.29^***^	−0.15	−0.09	−0.32^***^	−0.28^***^	−0.09	−0.22^**^	−0.20^*^	−0.29^***^	−0.12	−0.26^**^	−0.14	−0.29^***^	−0.35^***^	−0.38^***^	−0.22^**^	−0.32^***^	−0.33^***^
SL	−0.10	−0.11	−0.03	−0.16^*^	−0.07	0.01	−0.13	−0.01	−0.06	−0.01	−0.01	−0.10	−0.08	−0.13	−0.07	0.09	0.11	0.15
LL	−0.05	0.02	0.07	–	–	–	0.06	−0.05	−0.11	−0.05	−0.22^**^	−0.28^***^	−0.26^**^	−0.36^***^	−0.34^***^	−0.02	−0.10	−0.04
LW	−0.01	0.00	−0.02	–	–	–	0.14	−0.02	−0.06	−0.13	−0.24^**^	−0.32^***^	−0.11	−0.25^**^	−0.17^*^	0.02	−0.05	0.01
HI	0.04	0.14	0.08	0.23^**^	0.27^**^	0.15	0.03	0.04	−0.02	0.27^***^	0.18^*^	0.28^***^	0.06	0.00	−0.01	0.16	0.04	0.04
GPr	0.18^*^	0.07	0.10	0.10	0.08	−0.16	0.07	−0.01	0.03	−0.23^***^	−0.24^**^	−0.38^***^	−0.04	0.00	0.05	0.08	0.15	0.10

*Environment and trait definitions are as defined for Tables 1, 2, respectively*.

*Values are Pearson correlation coefficients and significance level indicated by*

* *p < 0.05*,

** *p < 0.01, and*

*** *p < 0.001*.

*–Indicates that the trait was not evaluated in the respective environment*.

The HTIs for TGW, GP, and Ret were strongly and positively correlated with TGW, GP, and Ret, respectively, across all delayed sown environments, indicating the strong reflection of genotypic differences for the traits into the HTIs. The HTIs were not related to the trait potentials (*r* = 0 in all cases, data not shown) or flowering time (*r* = 0; Table 4). Therefore, their relationship to the physical grain quality parameters under delayed sown conditions was independent of the effects of these confounding factors.

The HTIs showed moderate to strong correlations with the grain weight and size parameters. The HTIs were positively correlated with TW, TGW, GP, Ret, GWi, GTh, and SGW, and were negatively associated with Scr across delayed sown environments in both seasons (Table 4). TGW.HTI was correlated positively with GLe, whereas GP.HTI and Ret.HTI were correlated negatively with GLe (except Ret.HTI in MK19LS). Grain weight and size HTIs were generally better correlated with GWi and GTh than GLe, indicating that physical grain quality heat tolerance was more related to alteration of the grain's lateral dimensions.

Grain weight per spike showed only a few weak correlations with HTIs (Table 4), reflecting how it was better correlated with GNS than grain weight or size (Supplementary Table S7). Grain weight and size HTIs were consistently negatively correlated (weak to moderate) with GNS and HL across delayed sown environments. Therefore, genotypes with shorter heads and lower grain numbers (i.e., smaller sink size) maintained better grain weight and size under heat. Grain weight and size HTIs showed weak to moderate negative correlations with SFNS in 2018, reflecting the association of both traits with sink size.

Physiological traits generally had weak to moderate correlations with grain weight and size HTIs. Among the physiological characteristics, GFR and GFRmax showed relatively stronger and more consistent correlations with grain weight and size HTIs across delayed sown environments. The positive correlations of GFR and GFRmax with TGW.HTI, GP.HTI, and Ret.HTI across delayed sown environments indicate genotypes with better grain weight and size maintenance under heat had faster GFR. GFD and the related trait TIP showed weak and inconsistent correlations with TGW.HTI.

Relationships between WSC measures and grain weight and size maintenance appeared complex. WSCmax and MWSC had few weak to moderate negative correlations with grain weight and size HTIs. These correlations were opposite to expected if stem WSCs and absolute WSCs mobilisation contribute to maintaining grain weight and size under heat stress. HTI showed few negative correlations with WSCmin and a positive correlation with WSCME, demonstrating that genotypes with higher grain weight heat tolerance had a more efficient WSC mobilisation. WSCmin and WSCME also showed few weak to moderate correlations with grain size HTIs that were variable in direction. WSCmin was negatively correlated with GP.HTI and Ret.HTI in WH19LS and was positively correlated with Ret.HTI in WH19VLS. WSCME was positively correlated with grain size HTIs in WH19LS, while the reverse held in MK19LS. TGW.HTI and GP.HTI were positively correlated (weak to moderate) with NAUSC during grain-filling in WH19LS and MK19LS, indicating that stay-green contributed to better grain weight and size maintenance.

The penultimate leaf dimensions were negatively (weak to moderate) correlated with grain weight and grain size HTIs in WH19LS and WH19VLS. Therefore, smaller leaf sizes may have adaptive value in hot environments, probably through the heat avoidance mechanism. HI showed positive correlations with grain weight and size HTIs in WH18VLS and WH19LS, indicating that HI was favoured and may contribute to grain weight and size maintenance under heat conditions. GPr showed a weak positive correlation with TGW.HTI in WH18LS and moderate negative correlations with grain weight and size HTIs in WH19LS.

The relationship between the physical grain quality HTIs and other traits in PCA analyses were similar to those of the correlation tests. The PCA results indicate a substantial contribution of the GFR to the explained variation by the first two PCs and the consistent positive correlation between the physical grain quality HTIs and GFR across delayed sown/heat-stressed environments (Supplementary Figure S5).

**Figure 5:**
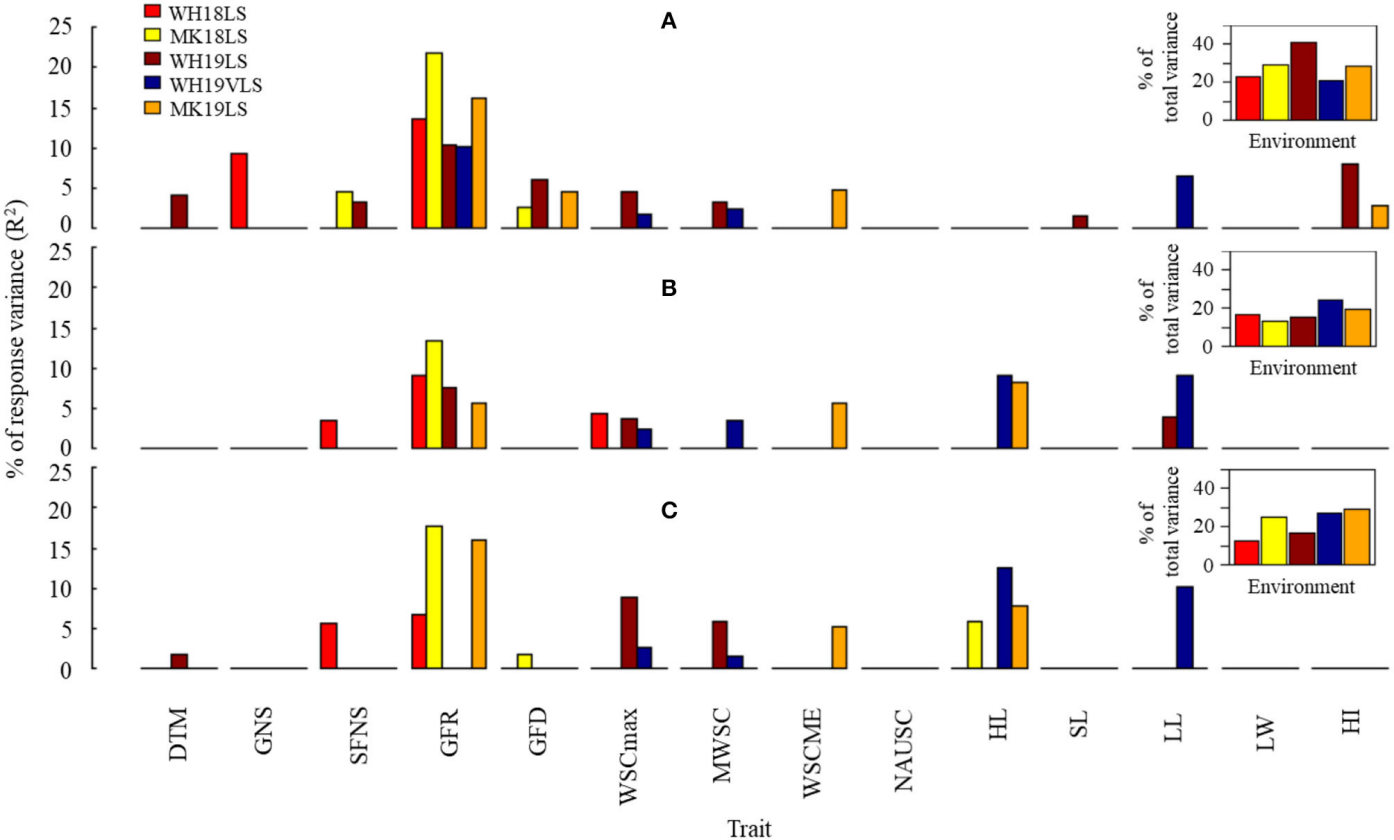
Estimated contribution (% of the variance, *R*^2^) of selected phenological, agro-morphological, and physiological traits to **(A)** thousand-grain weight heat tolerance (TGW.HTI), **(B)** grain plumpness heat tolerance (GP.HTI), and **(C)** retention heat tolerance (Ret.HTI) in WH18LS (light-red), MK18LS (yellow), WH19LS (dark-red), WH19VLS (navy-blue), and MK19LS (orange) environments. Insets show the total variability explained by the best significant model in each environment (*p* < *0.001*). Environment and trait definitions are as defined for Tables 1, 2, respectively.

The associations between TGW.HTI, GP.HTI, and Ret.HTI (as dependent variables) and selected phenological, agro-morphological, and physiological parameters (as independent variables) were tested in a stepwise multiple linear regression analysis for each delayed sown environment separately (except for WH19VLS) (Figure 5 and Supplementary Table S9). The included independent variables in the model accounted for 20.5–40.9%, 13.4–24.1%, and 12.4–29.1% of the variation for TGW.HTI, GP.HTI, and Ret.HTI, respectively, depending on the environment. GFR accounted for the most significant proportion of the contribution to HTIs (explained 10–22%, 6–13%, and 7–18% of variability for TGW.HTI, GP.HTI, and Ret.HTI, respectively). GFR is likely the most crucial trait in determining physical grain quality under heat stress. The exception was WH19VLS, in which GFR was not added to the model for grain size HTI (GP.HTI and Ret.HTI). HL and LL explained much of the variation in that environment. No HTI model included NAUSC or LW.

## Discussion

Late sowing with supplementary irrigation increased the probability of days with temperatures of above 30°C during grain filling in assessing a diverse set of barley genotypes for heat tolerance. GFR was the trait most highly and consistently associated with physical grain quality stability, indicating this trait as potentially linked to barley heat tolerance.

### Grain Weight, Plumpness, and Dimensions

The change in grain weight (TGW and SGW), plumpness (GP and Ret), and dimensions (GLe, GWi, and GTh) was not consistent with increasing average temperature and frequency and intensity of hot days (≥30°C) with later sowing (Table 1 and Figures 1, 2).

The lack of general grain weight response with delayed sowing may be explained to some degree by the compensatory relationship between GLe and GWi or GTh, even though the sites were irrigated to minimise the effects of drought stress during grain filling. Although not always significant, delayed sowing generally increased GLe (except in MK19LS, which was slightly lower) and reduced average GWi and GTh (except in WH18LS, which was slightly higher) (Figure 2). GWi and GTh tended to show lower reductions (or even an increase in the case of WH18LS) in delayed sown environments in which grain weight did not significantly differ from the respective NS. It appears that the GLe increased large enough (although not always significant; Figure 2) to compensate for the concomitant decrease in GWi and GTh, leading to an insignificant grain weight difference in those delayed sown environments with a respective NS. By contrast, in WH19VLS, the significant increase in GLe could not compensate for a large concomitant decrease in GWi and GTh. In MK19LS, the significant reduction in GLe and grain lateral dimensions (GWi and GTh) led to a significantly lower grain weight relative to the respective NS.

The contrasting response of grain length to grain width and thickness under high temperatures is also reported elsewhere. Watt (2020) observed an increase in grain length and a reduction in grain width and thickness in response to high field temperatures during grain-filling in barley. Li et al. (2018) observed a grain length increase, while the width was reduced in response to high temperature during grain-filling in wheat under late sown field heat-stressed conditions. Shi et al. (2016) reported the grain length and width effect of high night-time temperature under controlled conditions in rice. They concluded that grain length was not significantly changed in response to the heat treatment, whereas grain width was reduced considerably. By contrast, Aiqing et al. (2018) reported a more significant effect of heat stress on grain length than the width in wheat. Grain length growth occurs early in grain development in response to cell division and elongation in the caryopsis, whereas filling out (width/thickness) occurs later in grain development due to starch deposition in the developing endosperm. Tashiro and Wardlaw (1990) found that grain length was most sensitive to high temperature 7 days after flowering and was unaffected by the heat treatment, which commenced 8 days later. Grain width was highly responsive to high temperature at 12–20 days after anthesis. Therefore, the high-temperature effects on GLe and the grain lateral dimensions may have partly depended on the exact timing of heat stress in this study and the studies, as mentioned earlier.

The average grain plumpness (GP and Ret) trend across environments closely followed the trend in grain lateral dimensions (in particular GWi), reflecting that the width of the grain defines plumpness. Grain plumpness and lateral dimensions showed a more transparent relationship with hot days, heat intensity, and frequency during grain-filling (Table 1; Figures 1, 2).

Grain weight (TGW and SGW) and plumpness (GP, Ret; and Scr) were better correlated with GWi in environments with a higher degree of heat stress during grain-filling (Table 3 and Supplementary Table S7). Additionally, the HTI for TGW, GP, and Ret were more correlated with GWi and GTh than GLe (Table 4). These observations suggest that the observed genotypic variation for grain weight, plumpness, and size under delayed sown conditions (higher heat stress) was mainly related to the grain width response. The observed stronger environmental and genetic-by-environment effects for GWi than GLe reinforce this idea (Table 2). These results are similar to those of Li et al. (2018) who reported a stronger heat stress effect on GWi than GLe and a stronger correlation of grain weight response with grain width, but not grain length in wheat under field grain-filling heat stress conditions. In contrast, Aiqing et al. (2018) observed a larger effect of heat stress on grain length than grain width in response to 10 days heat stress commenced at anthesis under controlled environment conditions in wheat. They also found a positive correlation between grain weight and length, but not between grain weight and width.

Heat stress affects starch accumulation in grains by affecting enzyme activity in the starch synthesis pathway (Hawker and Jenner, 1993; Jenner, 1994; Keeling et al., 1994; Wallwork et al., 1998). It can also alter amylose and amylopectin deposition and the number and size of different types of starch granules (Bhullar and Jenner, 1985; Hurkman et al., 2003; Li et al., 2018). These factors could have contributed to the observed variation in grain weight and width among the genotypes in response to high temperatures in this study. For further discussion, see Section Grain Growth and Development.

### Grain Growth and Development

The final grain weight and grain size of barley is a function of the rate and the duration of grain-filling. This study's genotypic variation was larger for rate than duration (Table 2), with delayed sowing truncating the GFD and TIP, but enhancing GFR and GFRmax (Figure 2). These findings agree with several studies which demonstrated that genotype determines the GFR, while environmental factors (e.g., temperature) mainly affect the duration of the grain-filling period (Bruckner and Frohberg, 1987; Wardlaw and Moncur, 1995; Zahedi and Jenner, 2003; Dias and Lidon, 2009).

Grain weight (TGW and SGW) and plumpness (GP and Ret) were more highly correlated with GFR (and GFRmax) than GFD (and TIP) under heat stress conditions (Table 3 and Supplementary Table S7). Interestingly, GWi, which was the grain dimension best correlated with TGW, plumpness (GP and Ret), and HTIs (Tables 3, 4), showed a more consistent and much stronger correlation with GFR (and GFRmax) than GFD or TIP under delayed sown conditions (Figure 4 and Supplementary Table S7). Furthermore, TGW.HTIs, GP.HTIs, and Ret.HTIs were positively correlated with GFR (and GFRmax), but did not show a clear relationship with GFD and TIP (Table 4). Overall, these findings suggest that GFR and GFRmax plays an essential role in determining physical grain quality performance/maintenance under heat-stress conditions.

Research with wheat has similarly observed greater genotypic variation in GFR than GFD. In those studies, genotypes with higher grain weight (the most heat-tolerant) were those with higher GFR under high-temperature conditions in the field (Bruckner and Frohberg, 1987; Motzo et al., 1996) and in the controlled environmental conditions (Wardlaw and Moncur, 1995; Zahedi and Jenner, 2003; Dias and Lidon, 2009). Savin and Nicolas (1996) and Santiveri et al. (2002) reported similar relations between grain growth components and grain weight in barley and triticale. Additionally, Tashiro and Wardlaw (1989) observed that the greater tolerance of the rice (a subtropical cereal) grain to high temperature in comparison with wheat (a temperate cereal) was associated with an ability to increase the rate of grain-filling with increasing temperature. These studies reinforce GFR as a critical trait determining grain weight and size under high-temperature conditions. By contrast, a few studies found that grain weight was more closely associated with GFD than GFR under heat stress conditions (Stone and Nicolas, 1995; Shirdelmoghanloo et al., 2016a).

The finding provides an opportunity for breeding programs to enhance heat tolerance by selecting for higher GFRs. It also gives greater validity to studies on isolating biochemical and physiological characteristics within the grain that are sensitive to heat stress, especially as starch accounts for most of the grain's dry matter (~70%). The time-course of the grain growth is dominated by the kinetics of starch accumulation, with starch reduced proportionately more than protein by heat stress (Bhullar and Jenner, 1985). Many studies suggest that the supply of assimilates to the developing grains is not limiting the production of starch under high temperature in barley (MacLeod and Duffus, 1988; Wallwork et al., 1998) and wheat (Wardlaw et al., 1980; Jenner, 1994; Wardlaw and Wrigley, 1994). Stronger correlations between GFR and grain weight (TGW and SGW) and plumpness (GP and Ret, and also GWi and GLe) than between GFR and mobilised stem WSCs (MWSC and WSCME), and also lack of or even negative correlation between GFR and NAUSC under delayed sown conditions observed in this study may support that notion (Supplementary Table S7).

Under heat stress, starch synthesis may be the limiting factor. Wallwork et al. (1998) in barley and Jenner (1994) in wheat have shown reduced conversion of sucrose to starch in the endosperm under high temperatures. They related the reduction to the heat sensitivity of several enzymes in the starch synthesis pathway, particularly soluble starch synthase (SSS). While there was a general trend for high temperature to increase the average GFR across genotypes (Figure 2), some genotypes, however, appeared to show a reduction in GFR in this study (Supplementary Figure S3). With several isoforms of SSS identified in wheat endosperm (Denyer et al., 1995), isoforms of SSS may exist in barley, accounting for the observed genotypic variation response to heat stress for GFR. Another heat-tolerance mechanism reported is the recovery of lost enzyme activity following heat relief (Hawker and Jenner, 1993; Wallwork et al., 1998). The transfer of photosynthate from the crease vascular system of the grain into the endosperm could also possibly explain part of the temperature response among wheat genotypes (Wardlaw et al., 1995). A combination of the factors mentioned above may have contributed to the variation in grain growth rate under delayed sowing in this study.

Keeling et al. (1994) and Wallwork et al. (1998) suggest that increased GFR under high temperatures may be associated with increased rates of chemical reactions due to greater kinetic energy of molecules (e.g., enzymes and substrates) and more frequent collisions between them. High temperatures also increase the rate of import of photosynthate into the grain (Wardlaw et al., 1980), which may, at least in part, relate to the enhanced rate of grain-filling under high-temperature conditions.

The most noticeable effect of delayed sowing on grain growth was a premature truncation in grain-filling and is possibly due to the decline in activity of the starch biosynthetic system in barley (Wallwork et al., 1998) and wheat (Jenner, 1986). Alternatively, truncation in the GFD may be partly due to the grain's heat-induced accelerated senescence, limiting its development and ability to convert the delivered sugars into starch (Shirdelmoghanloo et al., 2016a). Furthermore, the earlier TIP and shorter GFD under high temperature may relate to temperature impacts on the expression of genes encoding starch synthesis enzymes and shortening the time to reach the maximum expression levels of the transcripts of the starch biosynthetic enzymes (Hurkman et al., 2003).

Rate and duration of grain-filling were negatively correlated across all environments in this study, with similar observations by Al-Karaki (2012), Bruckner and Frohberg (1987), and Wardlaw and Moncur (1995). The correlation was generally more robust in low-stressed environments compared with environments with a greater degree of heat stress (Supplementary Table S7). Environmental and genetic factors resulting in rapid GFR were associated with short GFD and compensation between both variables. Because the correlation between GFR and grain weight/plumpness was much greater than between GFD and grain weight/plumpness, particularly under heat conditions, selection for higher GFR and grain weight/plumpness without lengthening GFD may be plausible. Indeed, a high rate of grain growth over a shorter period of grain-filling (and an earlier inflection point) would be a desirable, risk-reducing pattern of grain-filling in barley in rain-fed environments. Such a breeding strategy has been suggested for other crops, such as wheat (Bruckner and Frohberg, 1987) and maize (Daynard and Kannenberg, 1976), in environments where temperature extremes can shorten the growing season.

### Green Leaf Area Duration

Scientific consensus suggests that grain-filling in wheat and barley is not source-limited under favourable growing conditions. Flag and penultimate leaf photosynthesis provide a significant proportion of assimilates to fill the developing grains in barley (Jebbouj and El Yousfi, 2009), but assimilate supply can become a limiting factor under stress (e.g., drought and heat) due to the loss of chlorophyll and, thus, reduced photosynthetic activity (Serrago et al., 2013).

This study used the penultimate leaf NAUSC during grain-filling to measure green leaf area retention (stay-green). Delayed sowing was associated with accelerated chlorophyll loss and reduced NAUSC (Figure 2). While the mechanisms of heat-induced chlorophyll loss resulting in a reduction in green leaf area and photosynthetic capacity are unknown, it could be associated with injury to thylakoid membranes that harbour chlorophyll. Harding et al. (1990) concluded that heat stress accelerates thylakoid component breakdown and induces a destabilising imbalance between component reaction rates. Ristic et al. (2007) found strong correlations between heat-induced injuries to thylakoids (and losses of PSII functionality) and chlorophyll loss.

Normalised area under the SPAD decline curve (NAUSC) was positively correlated with grain weight (TGW and SGW) and plumpness (GP and Ret, and also the grain lateral dimensions GWi and GTh) and duration (GFD and TIP) across environments (Table 3 and Supplementary Table S7). NAUSC was also correlated (albeit weakly) HTIs of the physical grain quality parameters in a few environments (Table 4). These results suggest that maintaining green leaf area for a more extended period during grain-filling (stay-green) may have contributed to some of the observed genotypic variations for grain weight and plumpness/size, particularly under delayed sown conditions. There are many reports of an association between stay-green, grain weight/size, and yield performance under drought or heat conditions in barley (Emebiri, 2013; Gous et al., 2016), wheat (Reynolds et al., 1994; Kumari et al., 2007; Lopes and Reynolds, 2012; Shirdelmoghanloo et al., 2016a) and sorghum (Borrell et al., 2014a,b). Furthermore, the higher grain weight of durum wheat stay-green mutants was related to the delayed loss of chlorophyll content, more extended photosynthetic competence (Spano et al., 2003), and longer expression of Rubisco activase, SSS, and glycine decarboxylase (Rampino et al., 2006). Further support for the idea that stay-green prolongs photosynthesis and GFD.

It is worth noting that NAUSC was negatively associated with SFNS across environments, indicating that stay-green has a role in higher spike fertility rate, particularly in environments exposed to a greater degree of heat stress (Supplementary Table S7).

### Stem Water-Soluble Carbohydrate

Water-soluble carbohydrates stored in stems also contribute to grain-filling in barley. Their contribution to final grain dry matter can increase under any stress which inhibits current assimilation, such as heat and drought (Austin et al., 1980; Blum et al., 1994; Blum, 1998; Ehdaie et al., 2006; Talukder et al., 2013). In this study, delayed sowing reduced stem WSC, which was associated with reduced stem volume (Figure 2) and WSCs accumulation (probably due to reduced photosynthesis; data not shown). Delayed sowing also reduced the absolute mobilised stem WSCs, but improved WSC mobilisation efficiency, particularly in 2018. Similar effects under heat or drought stress conditions have been reported in barley (Austin et al., 1980; Méndez et al., 2011) and wheat (Ehdaie et al., 2006; Talukder et al., 2013).

Maximum WSC and MWSC showed few negative correlations with plumpness (GP and Ret) and HTIs (Tables 3, 4). These correlations, however, were in the opposite directions to those expected if those parameters contribute to better physical grain quality performance under heat conditions. There were also few correlations between WSCME and the HTIs (Table 4). There was no clear relationship between stem WSCs content or the absolute stem WSCs remobilisation and the physical grain quality performance. However, grain weight (TGW and SGW) and plumpness (GP, Ret, and grain lateral dimensions) showed some positive correlations (weak to moderate) with WSCME (and in line with this negatively correlated with WSCmin) under delayed sown conditions, particularly in 2019. Genotypes with heavier and plumper grain potentials tended to have higher WSCME under heat-stress conditions. Furthermore, MWSC and WSCME showed positive correlations (albeit weak) with grain growth rate (GFR and GFRmax), particularly in delayed sown environments (Supplementary Table S7).

The results presented here suggest that stem reserves may not be an advantage unless plants can efficiently mobilise the reserves to the developing grains or convert delivered carbohydrates to starch in the grain (e.g., as a result of heat-stable SSS activity). It also suggests that the mobilised stem reserves may contribute to better physical grain quality performance *per se* through stabilising GFR, particularly under high-temperature conditions.

### The Trade-Off Between Grain Weight and Grain Plumpness With Grain Number

Grain number spike^−1^ (GNS) and HL showed a relatively consistent negative correlation with grain weight (TGW and SGW), grain plumpness (GP and Ret; and also grain dimensions) (Table 3 and Supplementary Table S7), and HTIs (Table 4). Sadras (2007) reported a similar trade-off between grain number and grain weight as observed in higher temperature conditions in this study. However, neither the physiological and genetic basis underlying this trade-off (Quintero et al., 2018) nor the environmental effect is well-understood.

Removal of florets before grain filling can increase the weight of the remaining grains (Fischer and HellieRisLambers, 1978; Calderini and Reynolds, 2000; Golan et al., 2019). A single amino acid substitution at a putative phosphorylation site in the *VRS1* gene in two-rowed barley *deficiens* mutants severely suppressed lateral florets and promoted grain weight (Sakuma et al., 2017). A recent study demonstrated a 12% improvement in grain weight without a negative effect on grain number due to increased levels of α-expansin in developing wheat grains by the ectopic expression of *TaExpA6* under control of a grain-specific gene promoter (Calderini et al., 2021). Therefore, the trade-off could stem from a growth limitation imposed by competition for an inadequate assimilate source (sink competition) or intrinsic limitations in grain weight potential.

The positive correlations between GNS with WSC content and mobilisation *per se* and for grain weight and plumpness with WSC mobilisation efficiency and stay-green (Table 3 and Supplementary Table S7) support the notion that assimilate supply probably had a role in determining grain weight, plumpness, and number. However, as those correlations were generally weak to moderate, other factors may have been in play, influencing the trade-off.

Quintero et al. (2018) found the trade-off to be very strong in environments with high temperatures and very low in cool favourable growing environments. In this study, the trade-off between GNS and HL with grain weight and plumpness was missing in the coolest growing environment (WH18NS). It was generally stronger in heat-stressed environments, suggesting a similar observation to Quintero et al. (2018). Interestingly, GFR was also negatively correlated with GNS, particularly in 2019 (Supplementary Table S7), indicating some compensating variability among the genotypes in both the GNS and GFR. A similar relationship was noted by Egli (2006) and Wu et al. (2018).

### Implications for Breeding

The present study identified heat-tolerant genotypes useful for barley breeding programs and identified traits for complementary selection criteria for heat tolerance. Heat tolerance, the physical grain quality potential, and heat escape (early flowering) all appeared to play a role in determining the barley genotypes' physical grain quality (TGW, GP, and Ret) assessed under the delayed sown conditions. However, they differed in their relative contribution, with heat tolerance and physical grain quality potential more important than heat escape. The accelerated development with later sowing may have reduced the contribution of heat escape as a mechanism.

Selection for high grain plumpness under low-stress conditions could enhance the breeding for heat stress tolerance, as genotypes with plump grain were less prone to small grain when exposed to higher temperatures under later sowing. However, the most efficient approach would be concurrent selection for factors affecting physical grain quality under heat stress. Selecting for high GFR, in particular, is likely to improve the future heat tolerance of barley as there was a highly significant positive correlation with physical grain quality parameters under heat stress conditions and with heat tolerance (Tables 3, 4, and Figures 4, 5). There was also strong genetic variation and very high heritability for GFR (Table 2).

## Conclusions

Responses to high temperature at the reproductive stage in barley have received less attention than other cereal crops such as wheat and rice. In this study, physical grain quality and a range of physiological, developmental, and agro-morphological traits were concurrently tested for their responses to natural heat events during reproductive stages of development in a diverse set of barley genotypes. Results presented here demonstrate a considerable heat impact on barley physical grain quality, but with a genetic variation.

Maximum progress in improving the physical quality of barley grain (weight and plumpness/size) in a future warmer climate could be achieved through combining physical grain quality potential, an appropriate developmental cycle (heat scape), and attributes associated with a high positive HTI (heat-tolerance) and physical grain quality *per se* under heat conditions. Generally, genotypes with better grain weight and plumpness performance *per se* and heat-tolerance under high temperatures tended to have high GFR, long grain-filling duration, long green leaf area retention, high WSC mobilisation efficiency, shorter heads with lower grain number, taller stature, smaller leaf size, and greater harvest index. GFR, however, had a significant role in determining barley grain weight and plumpness/size under grain-filling heat-stress conditions.

Additionally, results presented here suggest that stem WSC mobilisation and stay-green may contribute to better physical grain quality performance through their effect in stabilising GFR and duration, respectively. However, the stable GFR correlated with WSC mobilisation might be more influential in determining physical grain quality performance under heat stress conditions than grain-filling duration (and TIP) correlated with stay-green. The assimilates generated through ongoing photosynthesis and carbon losses due to other processes, such as respiration, were not estimated in this study. Further research considering these factors is required to get a better insight into contribution of the stem WSCs to physical grain quality performance/maintenance under heat conditions. The negative relationship between grain number and grain weight/plumpness performance and heat tolerance (and also GFR) suggest some level of compensating variability among the genotypes for these traits, particularly under heat-stress conditions. As heat tolerance and the associated traits presented here are challenging to measure in a breeding program, further work is required to detect genomic regions/genes controlling heat-tolerance traits, particularly GFR, and their use in heat-tolerance breeding through the delivery of validated DNA markers.

The irrigated conditions used in the current study may have favoured the expression of heat-tolerance mechanisms related to transpirational cooling. Furthermore, reductions in grain weight and plumpness/size in response to delayed sowing may stem from the accelerated development caused by exposing the crop to higher average temperature (in the non-stressful range) throughout the growing cycle, and also from an increased incidence of heat events at grain-filling. Transpirational cooling and accelerated development could have influenced the performance of the genotypes under delayed sowing. Therefore, it is essential to test heat-tolerant genotypes using in-field heat chambers when sown under normal rain-fed, not delayed sowing conditions.

## Data Availability Statement

The original contributions presented in the study are included in the article and Supplementary Materials. Further inquiries can be directed to the corresponding authors.

## Author Contributions

HS contributed to the project development and study design, conducted the experiments, contributed to the data collection and analysis, interpreted the data, and drafted the manuscript. KC made the experimental designs and undertook the data analysis, wrote the statistical methods section, and contributed to the data interpretation and preparation of the manuscript. BP contributed to the project development, study design, phenotyping, data interpretation, and manuscript preparation. TA conducted preliminary screening work, identified and provided germplasm, and contributed to the project development, study design, and manuscript preparation. SW contributed to the coordination, phenotyping, and preparation of the manuscript. HK and CH contributed to data interpretation and preparation of the manuscript. CL conceived of the study, contributed to its design and coordination, and contributed to data interpretation and preparation of the manuscript. All authors have read and approved the final version of the manuscript.

## Funding

This project was funded by the Grains Research and Development Corporation (GRDC; project UMU00049), with support from the Western Australian Department of Primary Industries and Regional Development (DPIRD) and Murdoch University.

## Conflict of Interest

The authors declare that the research was conducted in the absence of any commercial or financial relationships that could be construed as a potential conflict of interest.

## Publisher's Note

All claims expressed in this article are solely those of the authors and do not necessarily represent those of their affiliated organizations, or those of the publisher, the editors and the reviewers. Any product that may be evaluated in this article, or claim that may be made by its manufacturer, is not guaranteed or endorsed by the publisher.

## Abbreviations

DT49: Days to awn emergence (Z49)
DTM: Days to physiological maturity
GDD: Growing degree days (thermal time, GDD)
GFD: Grain-filling duration (GDD)
GFR: Average grain-filling rate (mg GDD^−1^, dry basis)
GFRmax: Maximum grain-filling rate (mg GDD^−1^, dry basis)
GLe: Grain length (mm)
GNS: Grain number spike^−1^ (number)
GP: Grain plumpness (% grain above 2.8 mm)
GP.HTI: Grain plumpness heat tolerance index
GPr: Grain protein content (%, dry basis)
GTh: Grain thickness (mm)
GWi: Grain width (mm)
GWS: Grain weight spike^−1^ (g)
HI: Harvest index (%)
HL: Head length (cm)
HTI: Heat tolerance index
LL: Penultimate leaf length (cm)
LS: Late sown
LW: Penultimate leaf width (cm)
MK: Muresk
MWSC: Absolute mobilised stem water-soluble carbohydrates during grain-filling (mg)
NAUSC: Normalised area under the penultimate leaf SPAD decline curve
NS: Normal sown
PCA: Principal component analysis
PC: Principal component
Ret: Retention (% grain above 2.5 mm)
Ret.HTI: Retention heat tolerance index
Scr: Screenings (% grain below 2.2 mm)
SFNS: Sterile floret number spike^−1^ (number)
SGW: Single grain weight (mg, dry basis)
SL: Stem length (cm)
SSS: Soluble starch synthase
TGW: Thousand-grain weight (g, dry basis)
TGW.HTI: Thousand-grain weight heat tolerance index
TIP: Thermal time from awn emergence to grain growth inflection point (GDD)
TW: Test weight (kg/hL)
VLS: Very late sown
WH: Wongan Hills
WSC: Water-soluble carbohydrates
WSCmax: Maximum stem water-soluble carbohydrates content (mg)
WSCME: Stem water-soluble carbohydrates mobilisation efficiency (%)
WSCmin: Minimum stem water-soluble carbohydrates content (mg).
