# Supplementary material for: Grain-Filling Rate Improves Physical Grain Quality in Barley Under Heat Stress Conditions During the Grain-Filling Period
Source: Front Plant Sci. 2022 May 13;13:858652. doi: 10.3389/fpls.2022.858652 (PMC9137397; doi:10.3389/fpls.2022.858652)
Supplement: Supplementary file 10 [file Data_Sheet_1.docx]

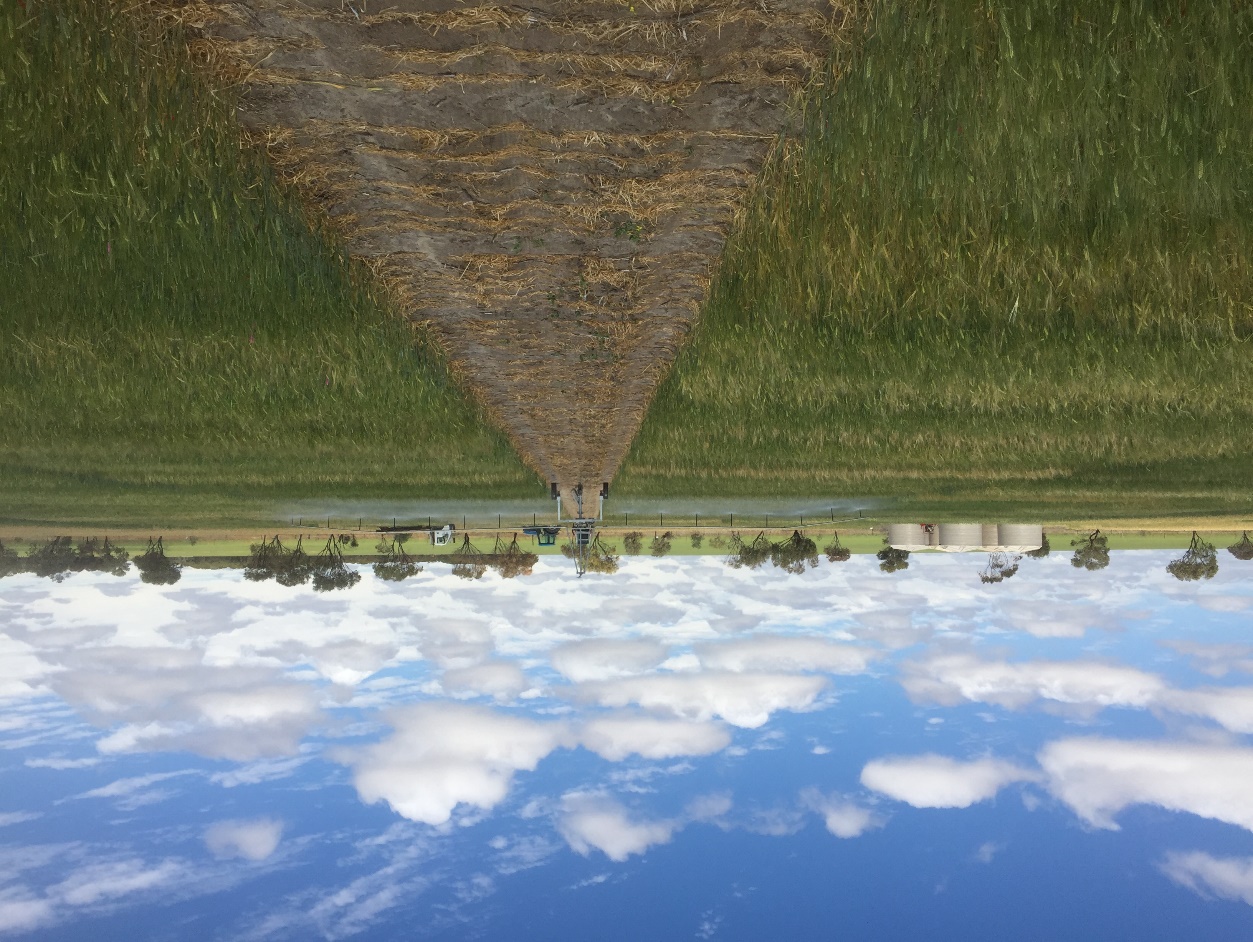


**Figure S1.** Irrigating a late sown trial at Wongan Hills using an overhead traveling irrigator.


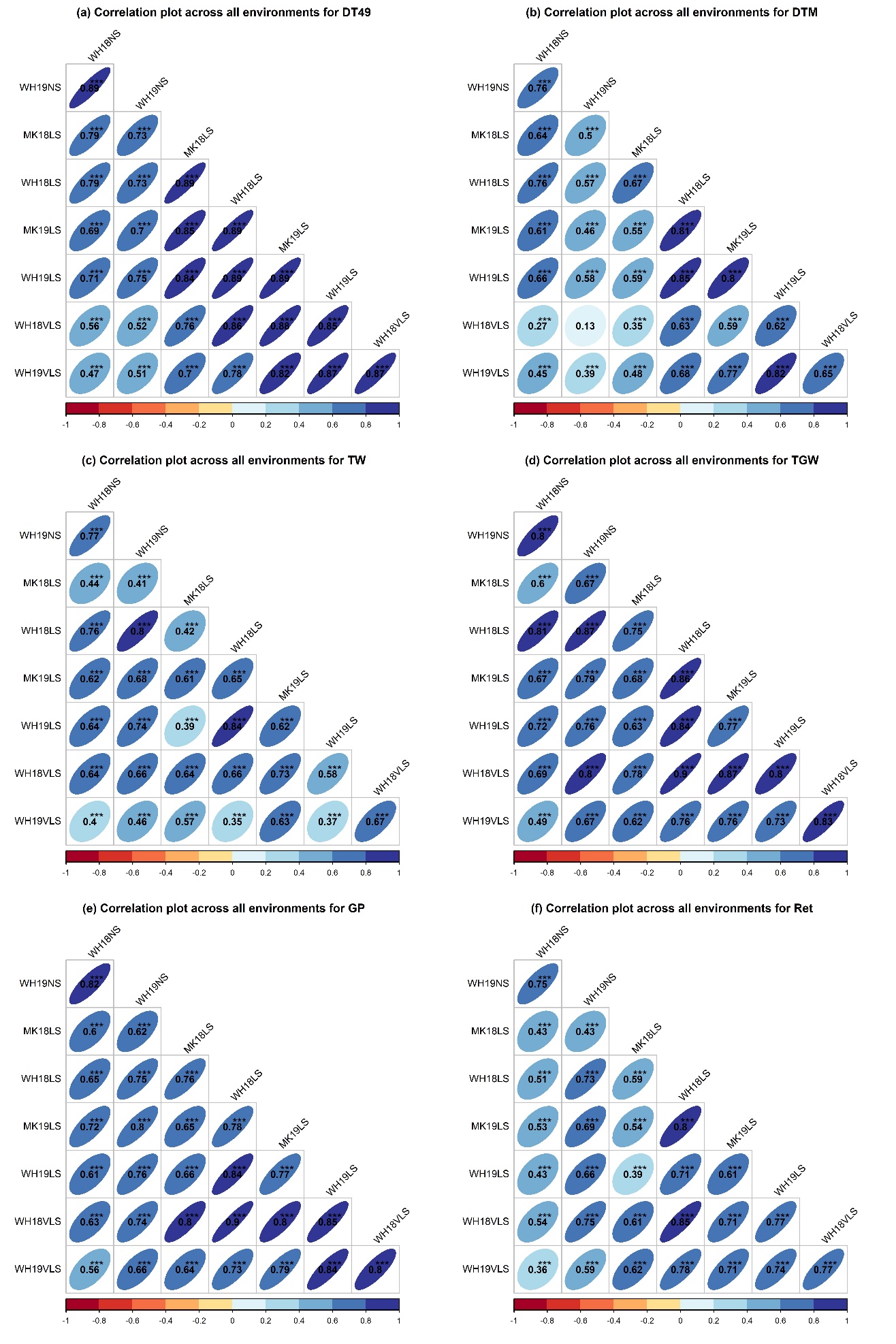


**Figure S2.** The correlation plots of each trait across environments. Environment and trait definitions are as defined in Tables 1 and 2, respectively. Values are Pearson correlation coefficients and significance level indicated by * $p <0.05$, ** $p < 0.01$, *** $p < 0.001$.


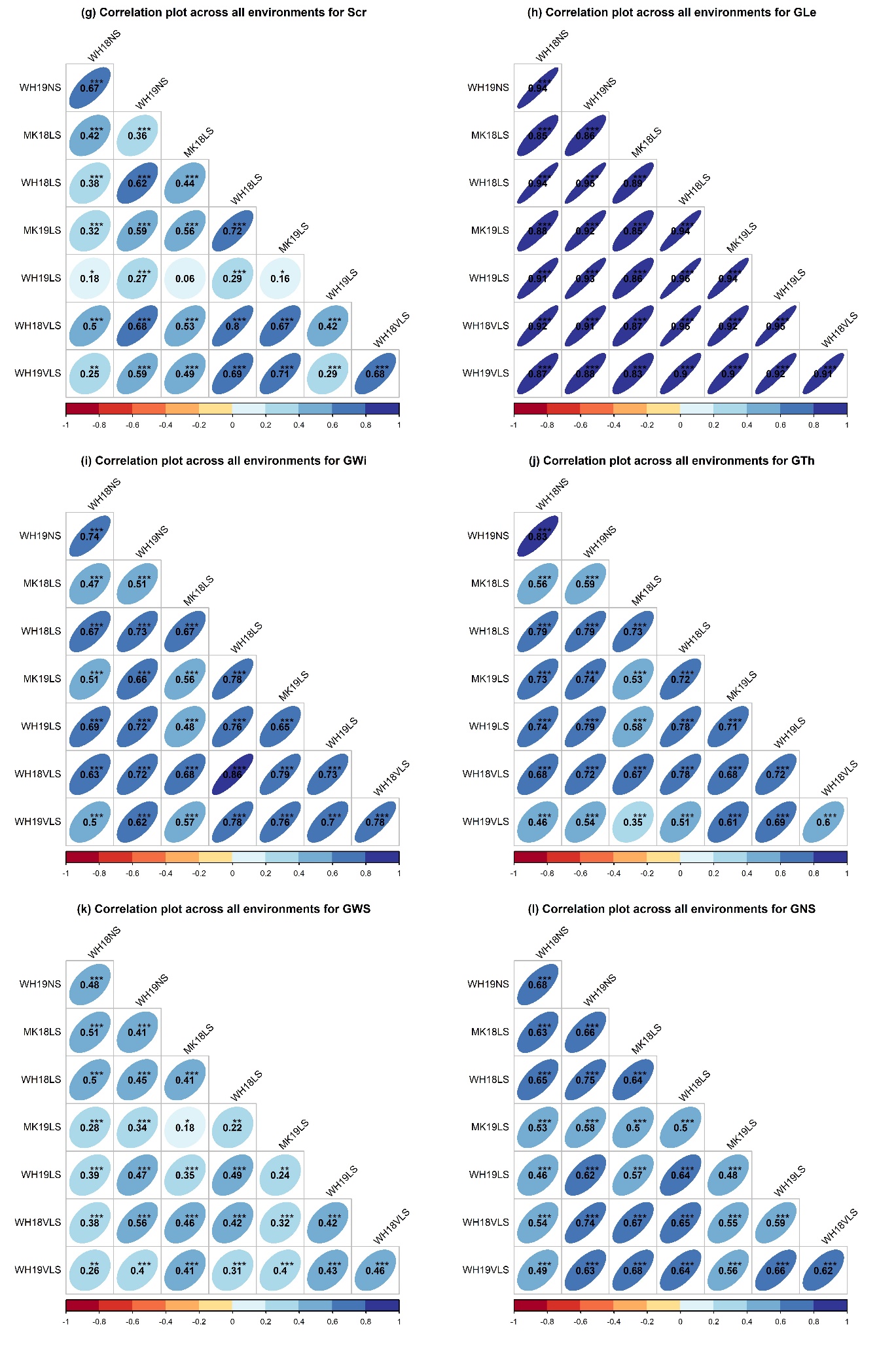


**Figure S2.** Continued


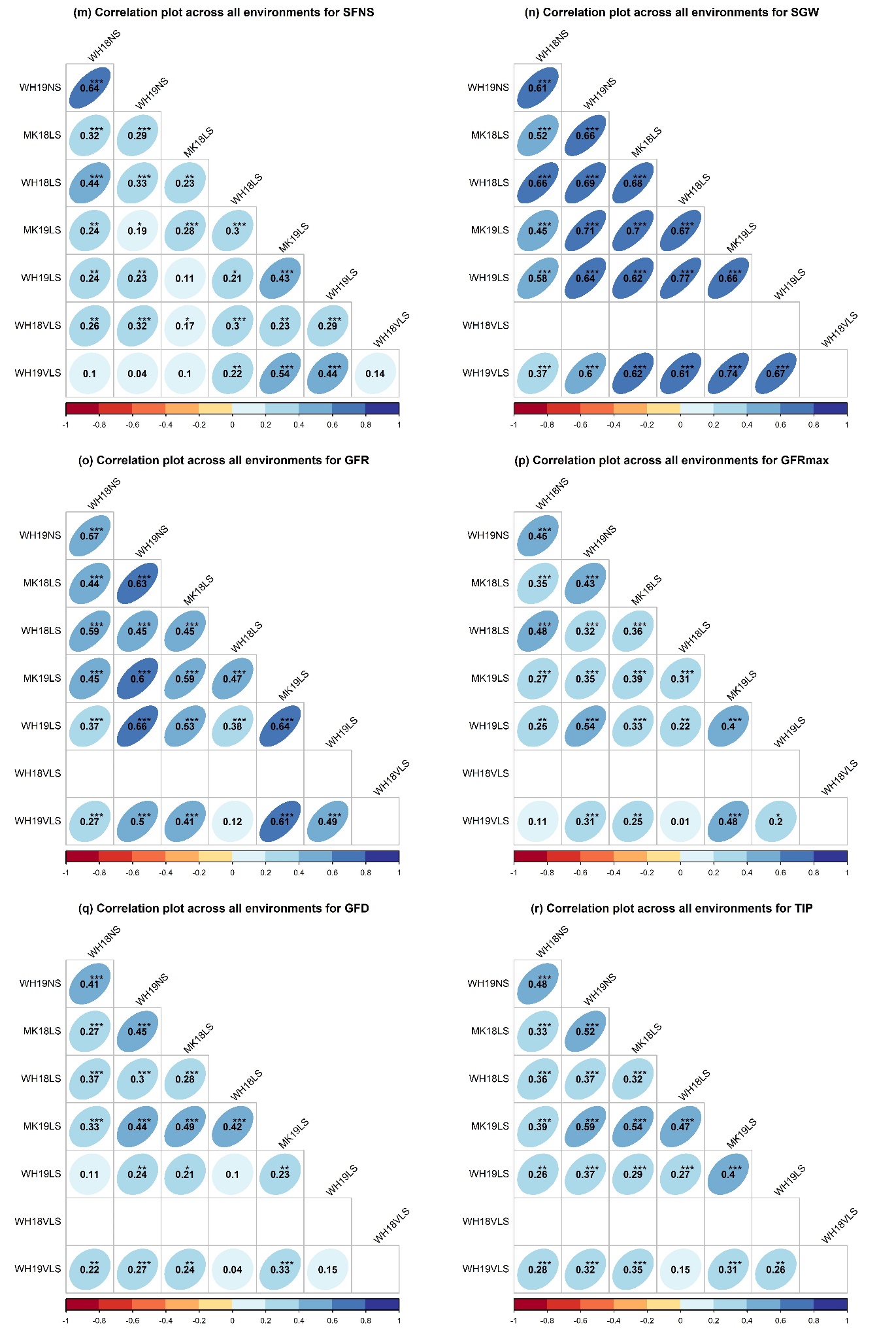


**Figure S2.** Continued


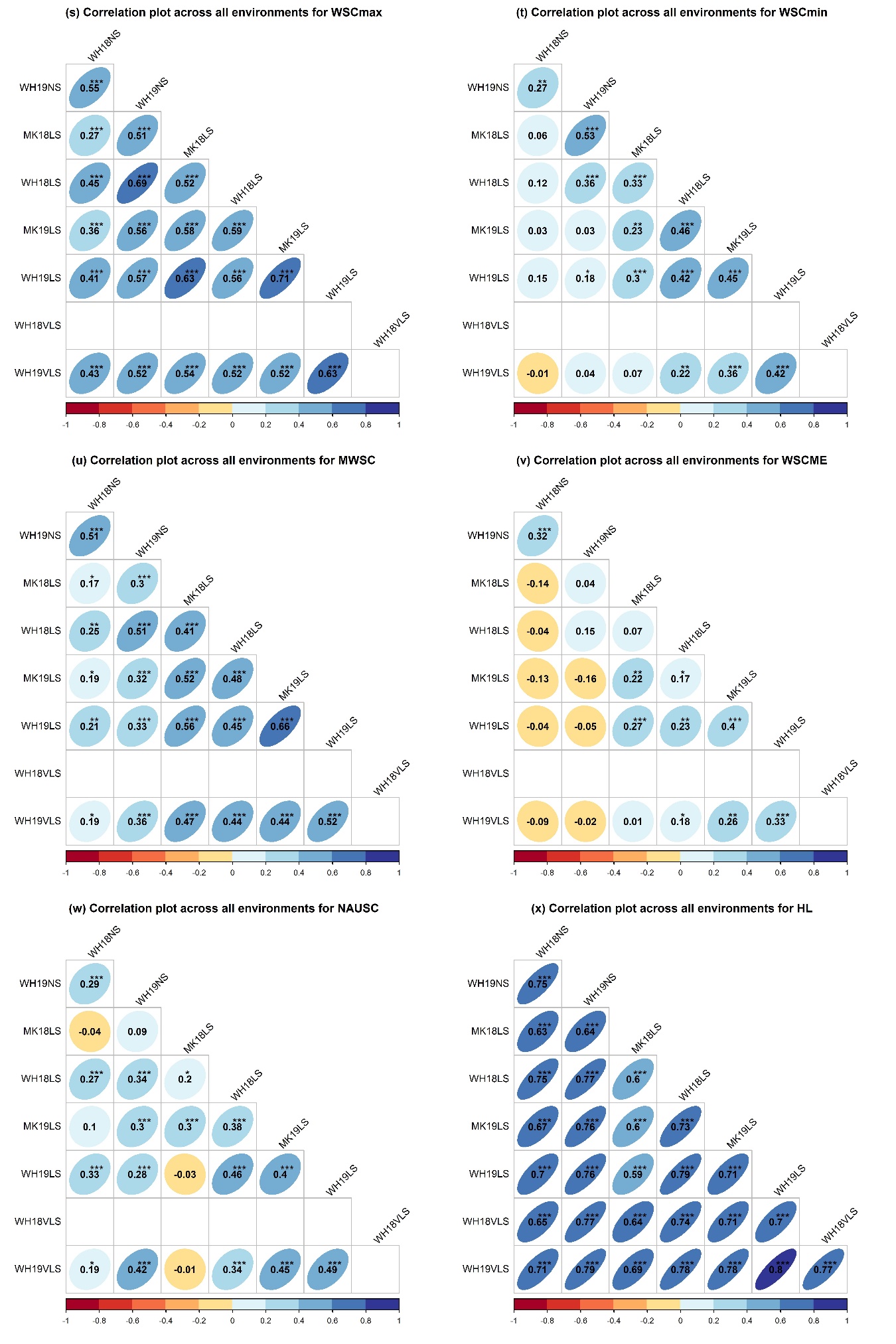


**Figure S2.** Continued


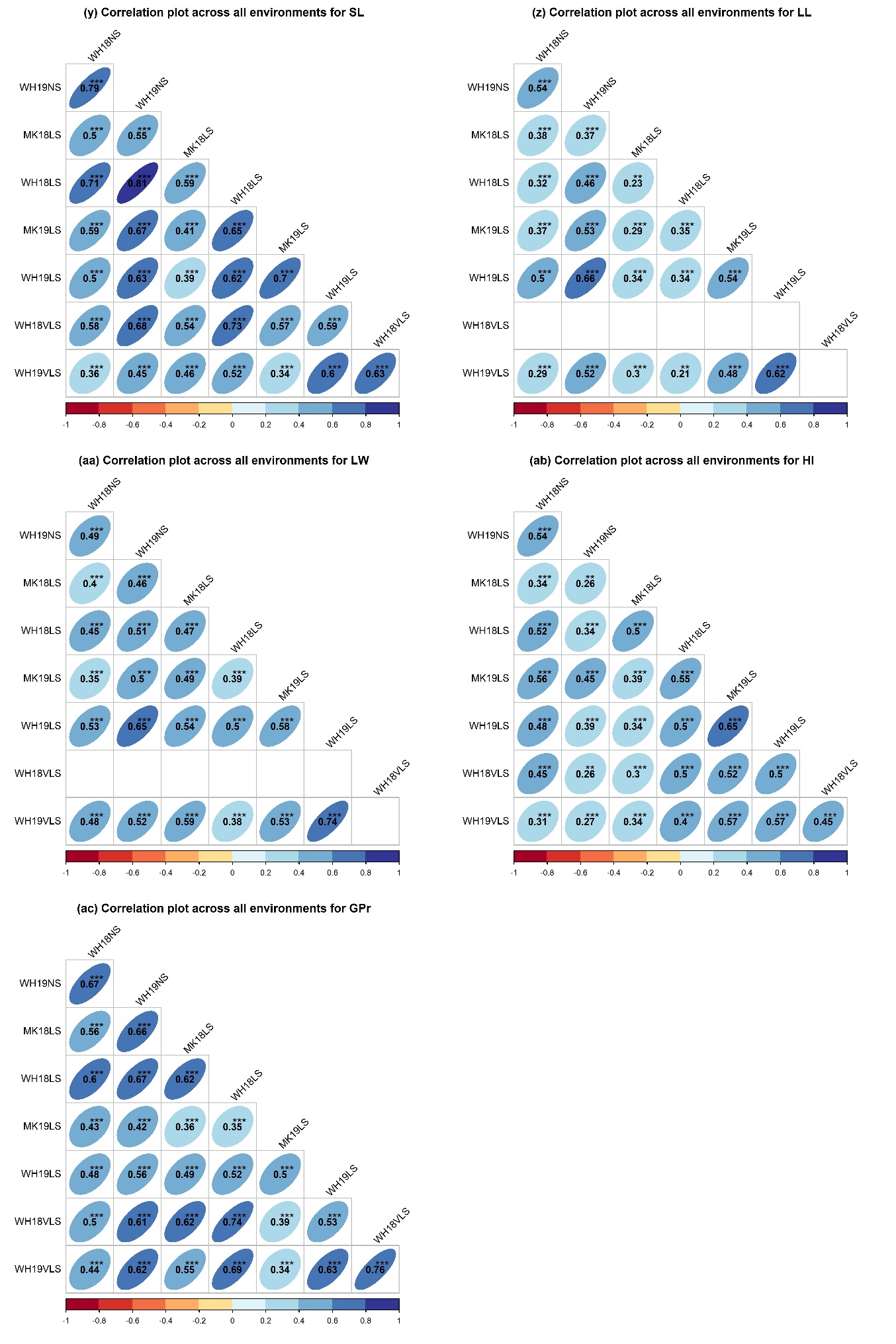


**Figure S2.** Continued


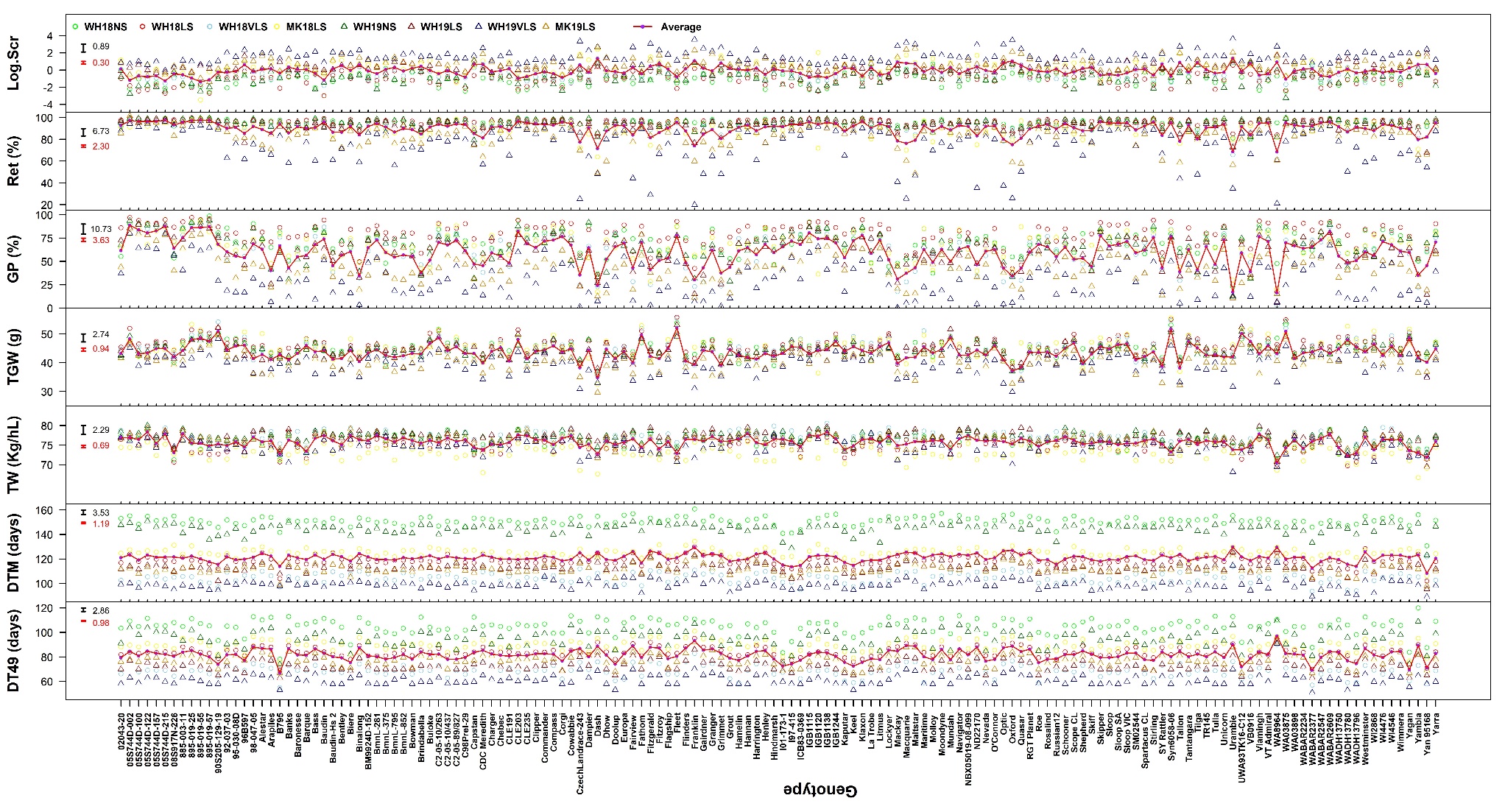


**Figure S3.** The eBLUEs of traits for each genotype (150 common genotypes across environments are shown) in WH18NS (light green circles), WH18LS (light red circles), WH18VLS (sky blue circles), MK18LS (yellow circles), WH19NS (dark green triangles), WH19LS (dark red triangles), WH19VLS (navy blue triangles) and MK19LS (orange triangles) environments. The red lines connect the means of genotypes across all environments. Environment and trait definitions are as defined for Tables 1 and 2, respectively. Black bars represent 2 × standard error of differences (SED) with α=0.05 for G × E effects, and red bars represent 2 × SED with α=0.05 for the means of genotypes across all environments. Grain growth components (SGW, GFR, GFRmax, GFD, and TIP), stem water soluble carbohydrates measures (WSCmax, WSCmin, MWSC, and WSCME), stay-green (NAUSC), and leaf dimensions (LL and LW) were not assessed in the WH18VLS environment.


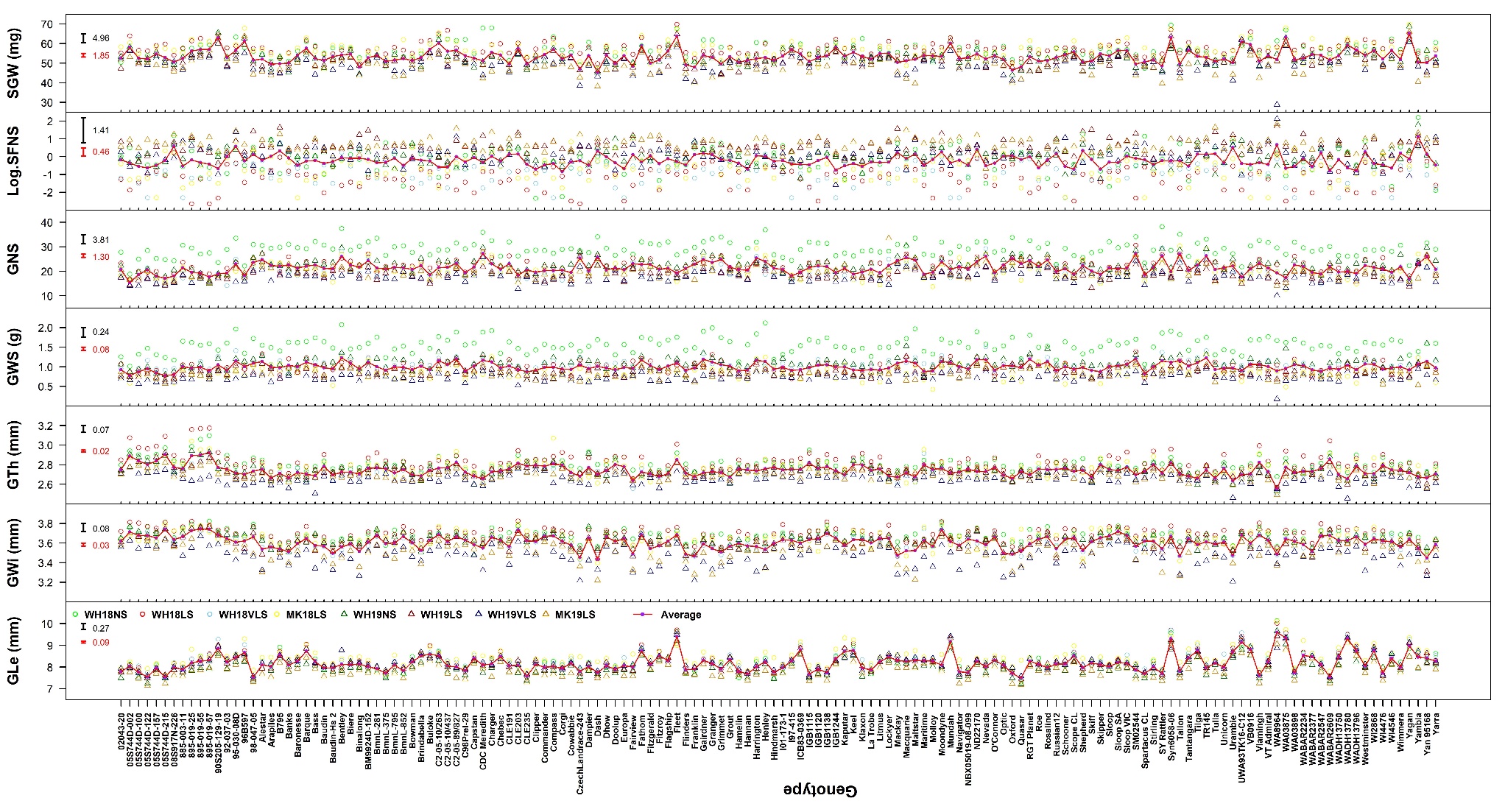


**Figure S3**. Continued


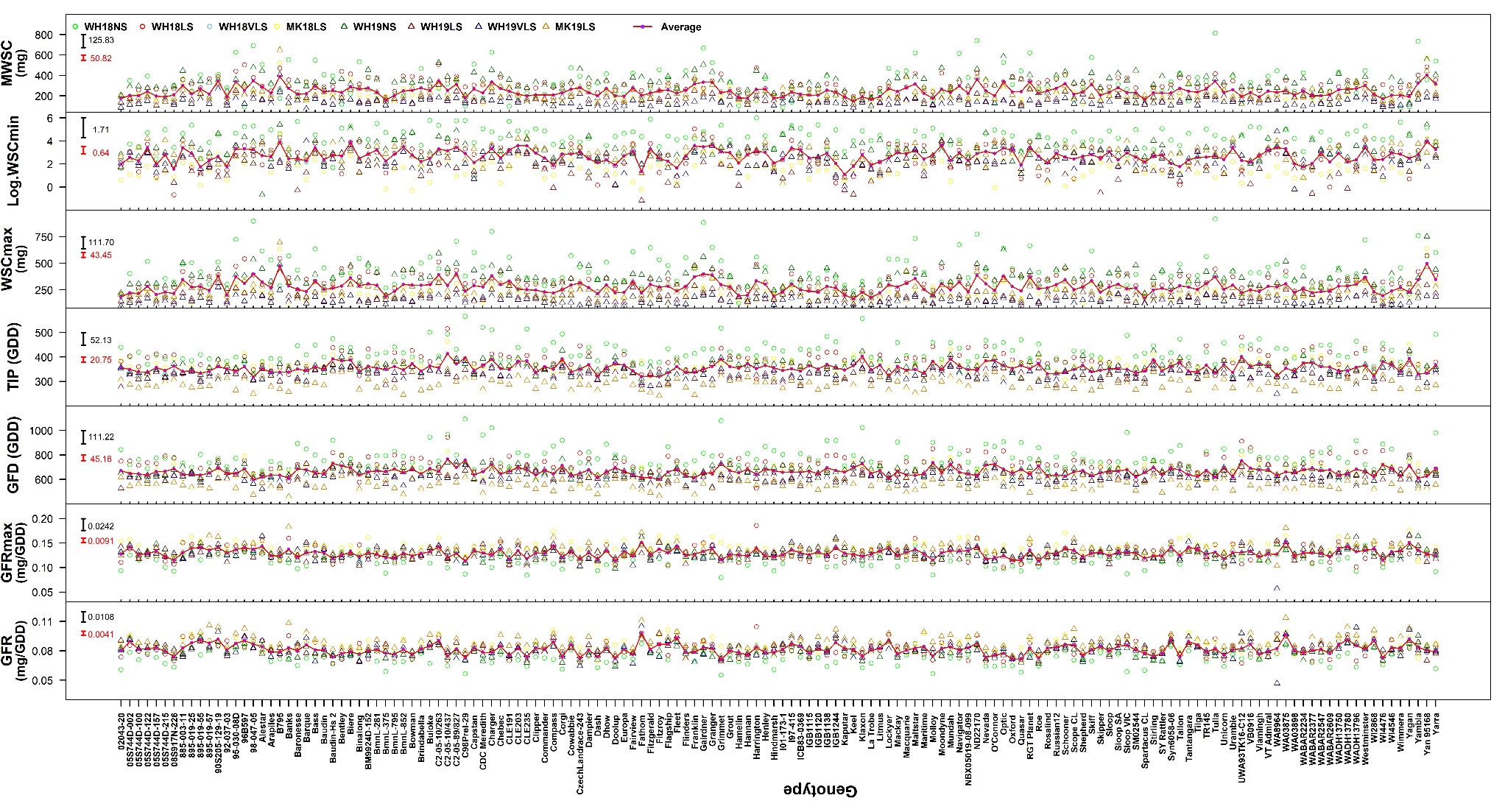


**Figure S3.** Continued


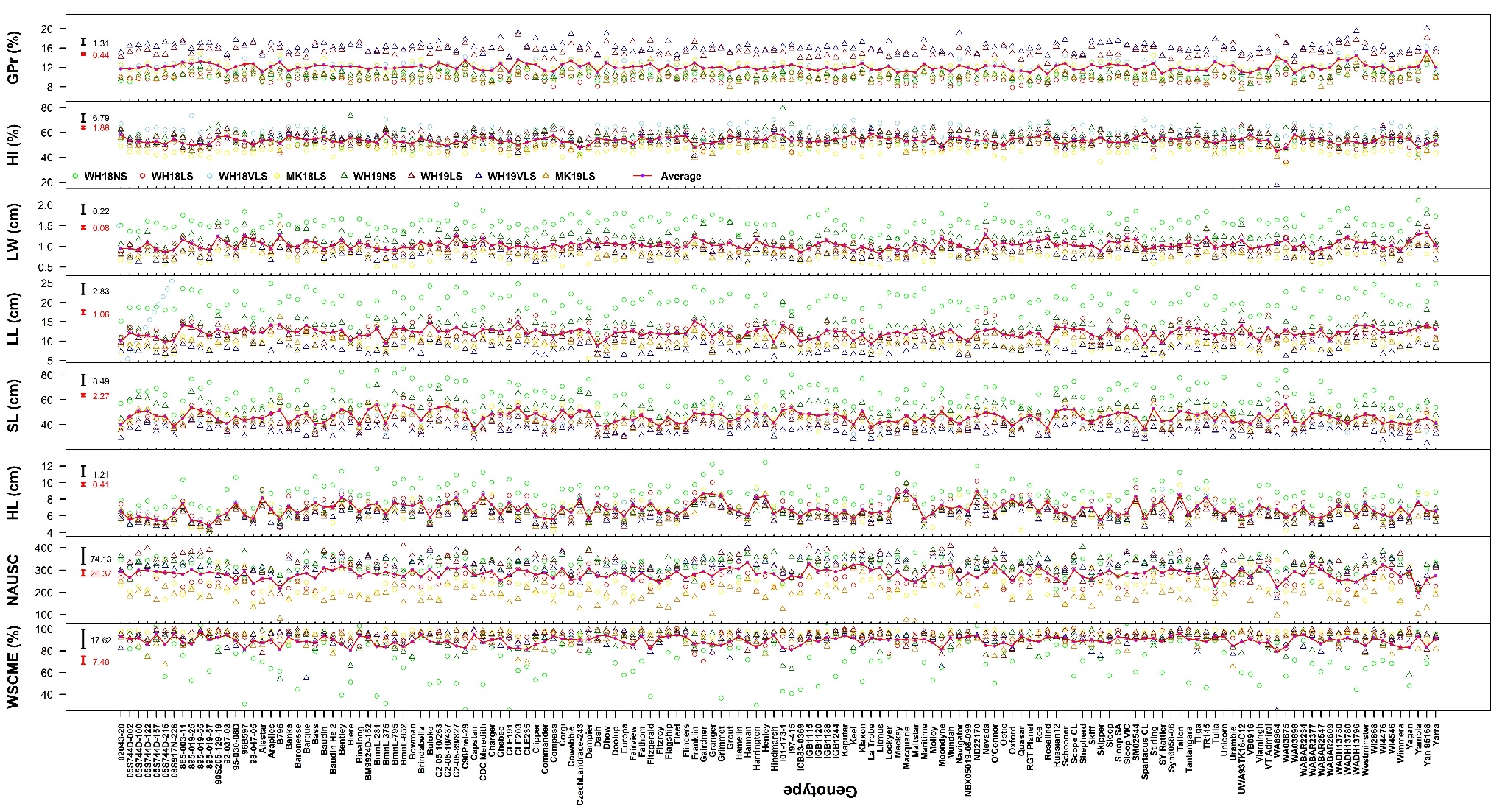


**Figure S3**. Continued


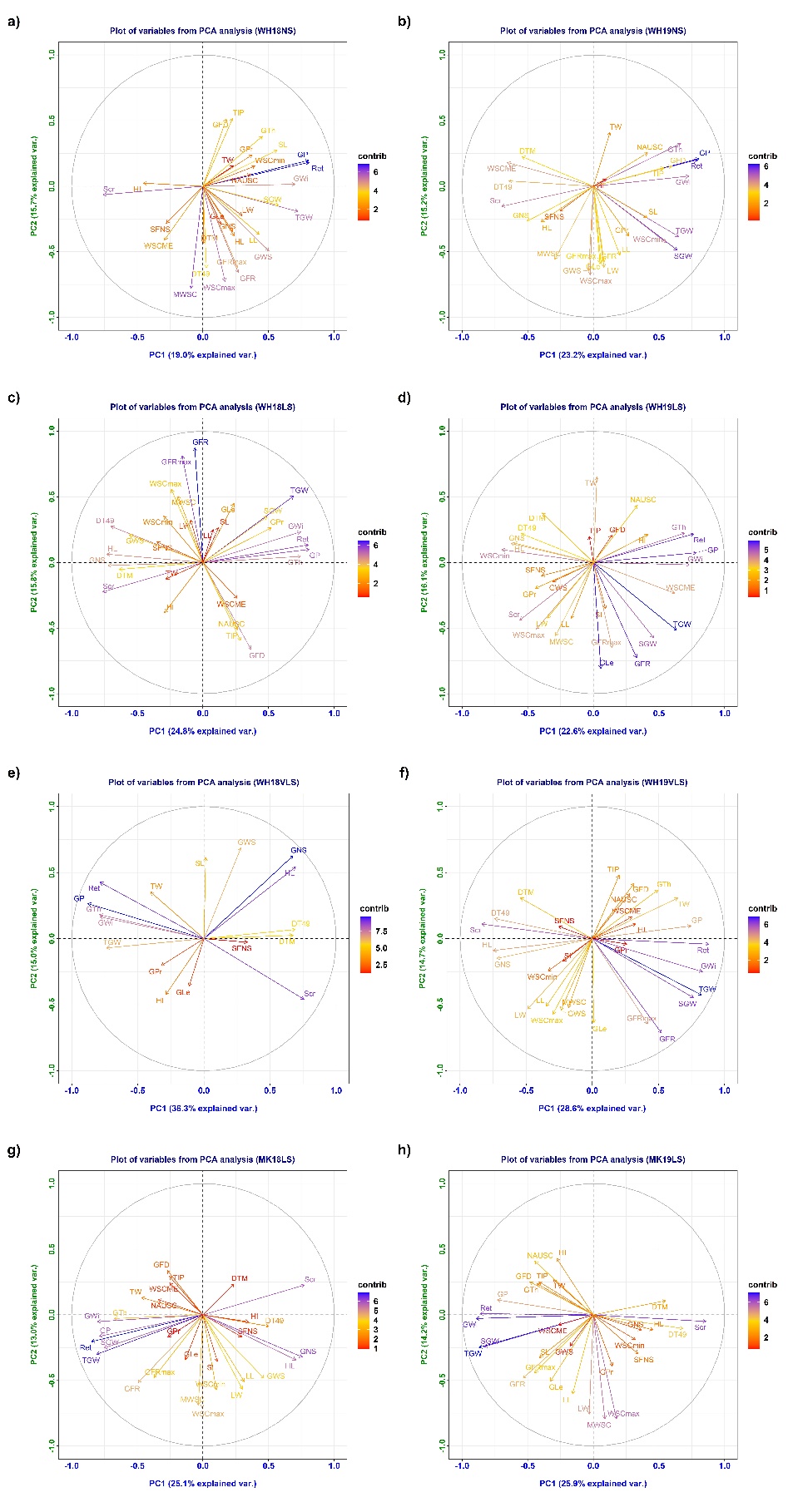


**Figure S4.** The plots of coordinates of the trait variables with the first two principal components 1 and 2 from the principal component analyses with all 8 environments. a) WH18NS, b) WH19NS, c) WH18LS, d) WH19LS, e) WH18VLS, f) WH19VLS, g) MK18LS, and h) MK19LS environments. Contib indicates the contribution of each trait to the first two principal components 1 and 2. Trait and environment definitions are as defined in Tables 1 and 2. Grain growth components (SGW, GFR, GFRmax, GFD, and TIP), stem water soluble carbohydrates measures (WSCmax, WSCmin, MWSC, and WSCME), stay-green (NAUSC), and leaf dimensions (LL and LW) were not assessed in the WH18VLS environment.


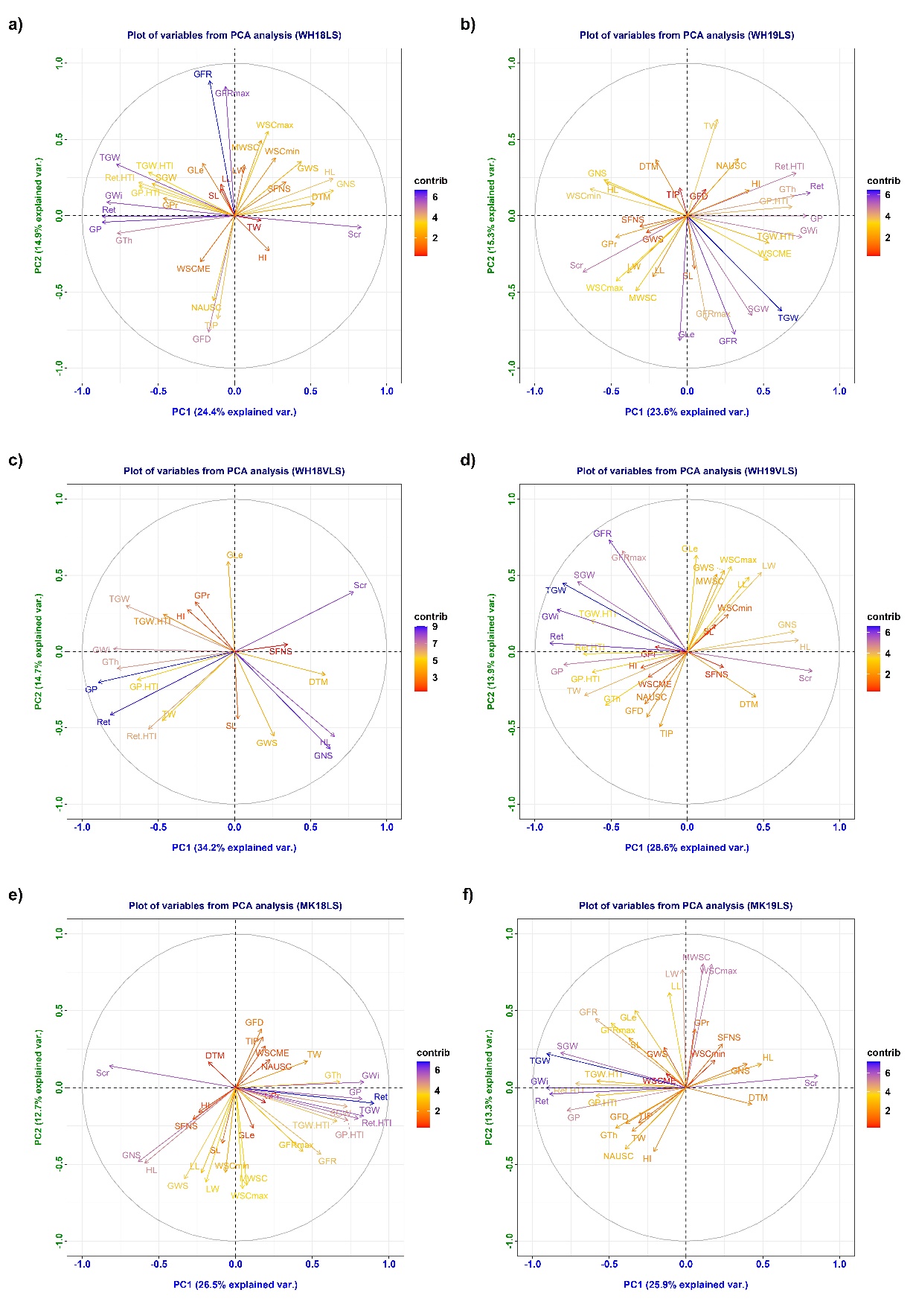


**Figure S5.** The plots of coordinates of the trait variables with the first two principal components 1 and 2 from the principal component analyses with the 6 delayed sown environments including heat tolerance indices of selected physical grain quality parameters (GP.HTI, Ret.HTI, and TGW.HTI). a) WH18LS, b) WH19LS, c) WH18VLS, d) WH19VLS, e) MK18LS, and f) MK19LS environments. Contib indicates the contribution of each trait to the first two principal components 1 and 2. Trait and environment definitions are as defined in Tables 1 and 2. Grain growth components (SGW, GFR, GFRmax, GFD, and TIP), stem water soluble carbohydrates measures (WSCmax, WSCmin, MWSC, and WSCME), stay-green (NAUSC), and leaf dimensions (LL and LW) were not assessed in the WH18VLS environment.
